# Supplementary material for: Innate Immune Response and Off-Target Mis-splicing Are Common Morpholino-Induced Side Effects in Xenopus
Source: Dev Cell. 2018 Mar 12;44(5):597–610.e10. doi: 10.1016/j.devcel.2018.01.022 (PMC5861998; doi:10.1016/j.devcel.2018.01.022)
Supplement: Document S1. Figures S1–S5 [file mmc1.pdf]

Developmental Cell, Volume *44*

Supplemental Information

Innate Immune Response and Off-Target Mis-splicing Are Common Morpholino-Induced Side Effects in *Xenopus*

George E. Gentsch, Thomas Spruce, Rita S. Monteiro, Nick D.L. Owens, Stephen R. Martin, and James C. Smith

Legends for Supplemental Figures

Figure S1. Generation of the Double Heterozygous Line for *Brachyury* paralogues *t* and *t2*, Related to Figure 1

(A) Scheme to generate the $t^{e1.2D/+}t2^{e3.7D/+}$ ($t^{-/+}t2^{-/+}$) *X. tropicalis* line. (B,F) TALEN design for *t* or *t2* mutagenesis and positions of MOs blocking donor splice site (MO_{splice}) or translation initiation site (MO_{transl}) of the corresponding transcript. (C,G) TALEN-induced mutation rate at the targeted *Sac*I or *EcoR*I site as estimated by the partial restriction digest of specific PCR amplicons. (D) Sanger sequencing summary of generated indels in exon 1 of *t*. (E) Morphological defects caused by TALEN-induced *t* mutations at late tailbud stage. Scale bar, 0.5 mm. (H) Western blot of injected wild-type and mutant *t* or *t2* constructs tagged either N- or C-terminally with *HA*. The detection of exogenous myc (as part of the injected *fam83g-myc* mRNA) and endogenous α-tubulin were used as controls for injection/translation efficiency and gel electrophoresis loading, respectively. (I) Mutant *Brachyury* constructs failed to disrupt gastrulation. Scale bar, 0.25 mm.

Figure S2. *Brachyury* KO and KD Embryos Are Morphologically Very Similar, Related to Figure 2

(A) Gross morphological comparison between different conditions of the KD and KO experiment at the indicated tailbud stages. Uninjected (uni) and control MO (cMO)-injected embryos were controls for the *t/t2* morphants (*t/t2* MO). Wild-type (wt) embryos were siblings of the mutant embryos (hetero- and homozygous for the mutant *t* and *t2* allele, *t/t2* het and *t/t2* KO). Scale bar, 0.5 mm. (B) Comparison of low fragment count gene transcripts to estimate minimal fragment count required for calling reliable fold changes. Genes that averaged <7 fragments between cMO-injected and uninjected and heterozygous and wild-type embryos over tailbud stage 26 and 34 were excluded due to a higher degree of spurious fold changes.

Figure S3. Increased Transcription of *tp53* Depending on GC Content of MO Does Not Cause More Apoptosis, Related to Figure 3 and 4

(A) TUNEL assay on morphants and sibling embryos from double heterozygous $t^{-/+}t2^{+/-}$ parents. DNase-treated wild-type embryos were used as positive controls. (B) Single WMISH for *tp53 and* multi-probe WMISH for various mesoderm cell lineage and

derivative markers (*cav1*, notochord; *hoxd8*, pronephros; *myh6*, heart; *tal1*, ventral blood island; *tbx6*, paraxial mesoderm) of late tailbud embryos injected with single MOs or tracer sulforhodamine-dextran. Scale bar, 0.5 mm.

Figure S4. Specific MOs of the *t/t2* MO Cocktail Cause Off-Target Splicing Defects, Related to Figure 5

(A,C) Superimposed Sashimi plot of *abi1* and *bloc1s4* transcripts whose splicing was perturbed by the injected *t/t2* MO mix. Canonical and alternative splicing are shown with solid and dashed lines, respectively. Blocked splice sites containing matches of ≥8 consecutive bases with a specific MO are shown as alignments. Canonical Watson-Crick and non-canonical wobble base pairing are marked as vertical bar and colon, respectively. (B,D) RT-qPCR (n = 4, mean ± SD) confirmed that specific MOs of the *t/t2* MO mix were responsible for mis-splicing. The fold change ($\log_2$ scale) of transcript levels and alternative splicing between exon 7 and 11 (*abi1*) and exon 4 and 6 (*bloc1s4*) are shown as filled and solid bars, respectively. Two-tailed t-test: *, p ≤0.1; **, p ≤0.01. See Figure 3D and Key Resources Table for the design of RT-qPCR primers.

Figure S5. Temperature and MO Dosage Effects on *Brachyury* Phenotype and Immune Response Related Gene Transcription, Related to Figure 7

(A) WMISH for *tp53* of late tailbud embryos injected with 4.5 or 18 ng of the *t/t2* MO mix and developed at 22 ºC or 28.5ºC. *, Remark: Increasing incubation temperature also slightly up-regulated *tp53* in the absence of any MO (confirmed by RT-qPCR, data not shown). (B) Multi-probe WMISH for various mesoderm cell lineage and derivative markers (*cav1*, notochord; *hoxd8*, pronephros; *myh6*, heart; *tal1*, ventral blood island; *tbx6*, paraxial mesoderm) and single WMISH for *tp53, tp53inp1* and *c3ar1* of mid-tailbud (stage 26) and late tailbud embryos (stage 34) injected with 6 or 18 ng of the *t/t2* MO mix. White arrowheads point to the expression domains of *tbx6* and *cav1* that were not maintained in embryos without functional Brachyury. (C) WMISH for *tp53inp1* on wild-type (or *t/t2* heterozygous) and *t/t2* null mutant embryos as well as embryos injected with 1 and 3 ng MO. The embryos were developed to late tailbud stage 34 at 25-26ºC. Scale bar, 0.5 mm.

Figure S1

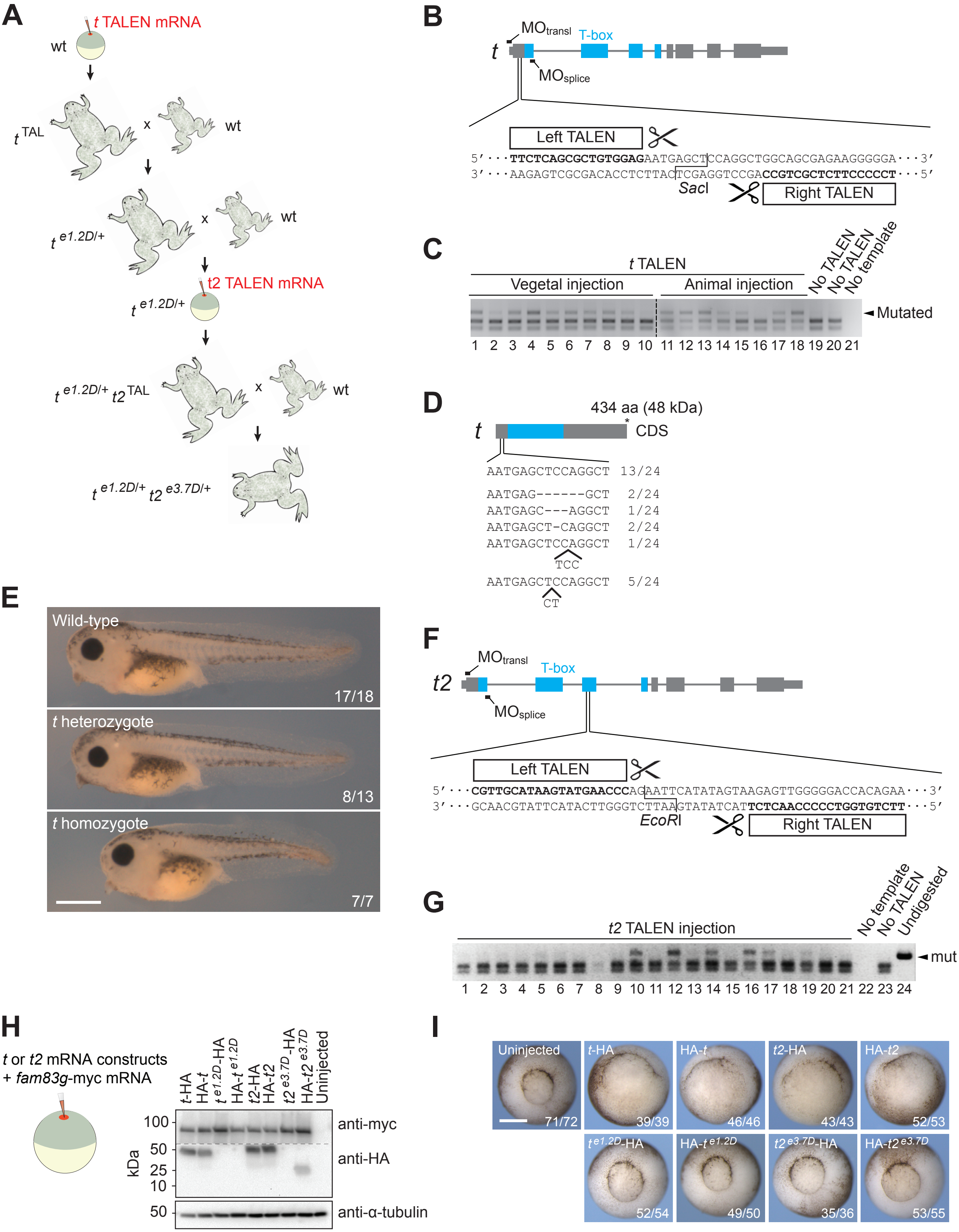

A
t TALEN mRNA
wt
t TAL
x
wt
t e1.2D/+
x
wt
t2 TALEN mRNA
t e1.2D/+
t e1.2D/+ t2 TAL
x
wt
t e1.2D/+ t2 e3.7D/+
B
MOtransl
T-box
t
MOsplice
Left TALEN
5′···TTCTCAGCGCTGTGGAGAATGAGCTCCAGGCTGGCAGCGAGAAGGGGGA···3′
3′···AAGAGTCGCGACACCTCTTACTCGAGGTCCGACCGTCGCTCTTCCCCCT···5′
SacI
Right TALEN
C
t TALEN
Vegetal injection
Animal injection
No TALEN
No TALEN
No template
Mutated
1 2 3 4 5 6 7 8 9 10 11 12 13 14 15 16 17 18 19 20 21
D
434 aa (48 kDa)
t
CDS
AATGAGCTCCAGGCT 13/24
AATGAG------GCT 2/24
AATGAGC---AGGCT 1/24
AATGAGCT-CAGGCT 2/24
AATGAGCTCCAGGCT 1/24
TCC
AATGAGCTCCAGGCT 5/24
CT
E
Wild-type
17/18
t heterozygote
8/13
t homozygote
7/7
F
MOtransl
T-box
t2
MOsplice
Left TALEN
5′···CGTTGCATAAGTATGAACCCAGAATTCATATAGTAAGAGTTGGGGGACCACAGAA···3′
3′···GCAACGTATTCATACTTGGGTCTTAAGTATATCATTCTCAACCCCCTGGTGTCTT···5′
EcoRI
Right TALEN
G
t2 TALEN injection
No template
No TALEN
Undigested
mut
1 2 3 4 5 6 7 8 9 10 11 12 13 14 15 16 17 18 19 20 21 22 23 24
H
t or t2 mRNA constructs
+ fam83g-myc mRNA
t-HA
HA-t
t e1.2D-HA
HA-t e1.2D
t2-HA
HA-t2
t2 e3.7D-HA
HA-t2 e3.7D
Uninjected
100
50
25
10
50
kDa
anti-myc
anti-HA
anti-α-tubulin
I
Uninjected
71/72
t-HA
39/39
HA-t
46/46
t2-HA
43/43
HA-t2
52/53
t e1.2D-HA
52/54
HA-t e1.2D
49/50
t2 e3.7D-HA
35/36
HA-t2 e3.7D
53/55

Figure S2

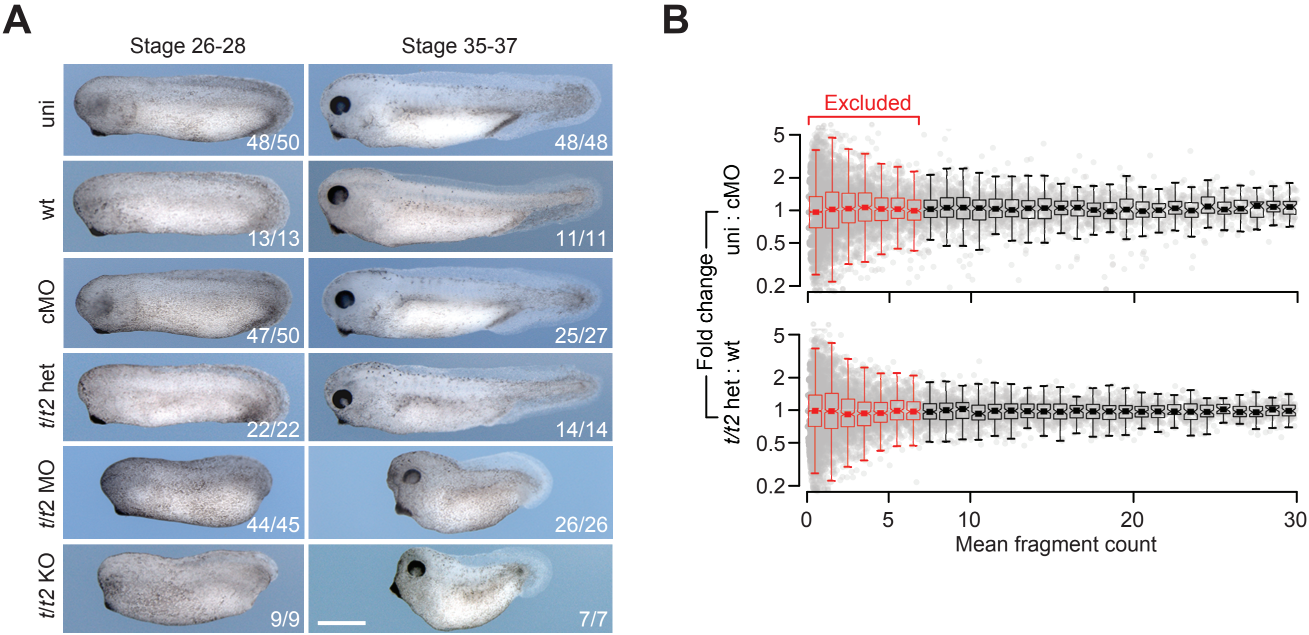
A
Stage 26-28
Stage 35-37
uni
wt
cMO
t/t2 het
t/t2 MO
t/t2 KO
48/50
48/48
13/13
11/11
47/50
25/27
22/22
14/14
44/45
26/26
9/9
7/7
B
Excluded
Fold change
uni : cMO
t/t2 het : wt
5
2
1
0.5
0.2
0
5
10
20
30
Mean fragment count

Figure S3

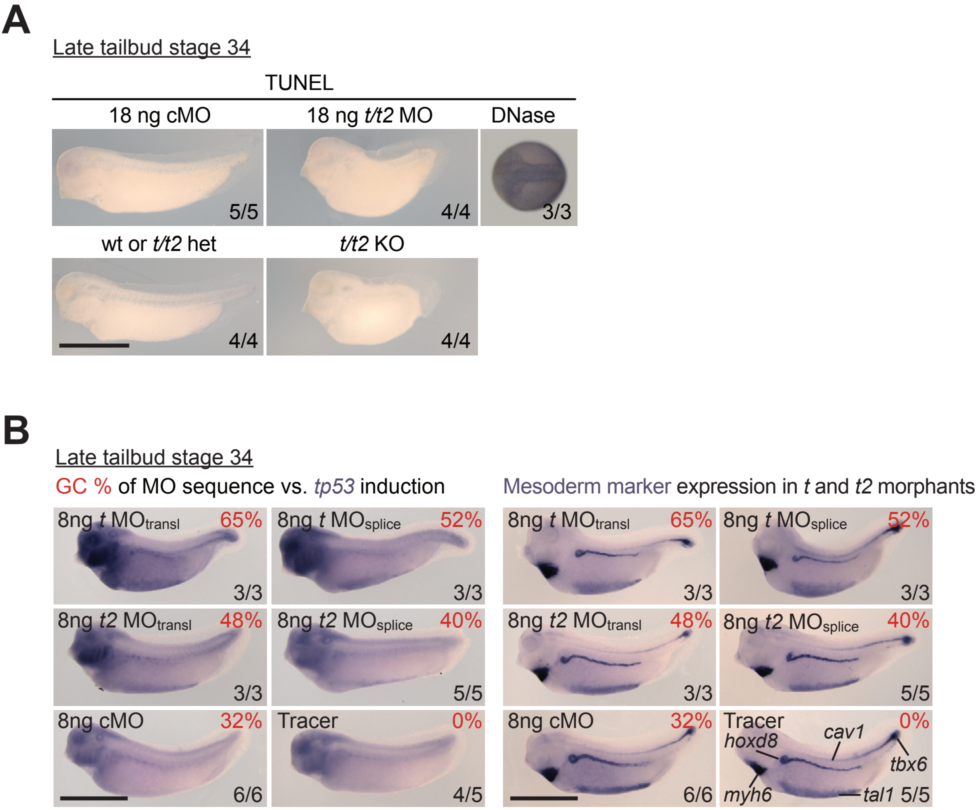
A
Late tailbud stage 34
TUNEL
18 ng cMO
18 ng t/t2 MO
DNase
5/5
4/4
3/3
wt or t/t2 het
t/t2 KO
4/4
4/4
B
Late tailbud stage 34
GC % of MO sequence vs. tp53 induction
Mesoderm marker expression in t and t2 morphants
8ng t MOtransl 65%
8ng t MOsplice 52%
8ng t2 MOtransl 48%
8ng t2 MOsplice 40%
8ng cMO 32%
Tracer 0%
3/3
3/3
3/3
5/5
6/6
4/5
8ng t MOtransl 65%
8ng t MOsplice 52%
8ng t2 MOtransl 48%
8ng t2 MOsplice 40%
8ng cMO 32%
Tracer 0%
hoxd8
cav1
tbx6
myh6
tal1
3/3
3/3
3/3
5/5
6/6
5/5

Figure S4

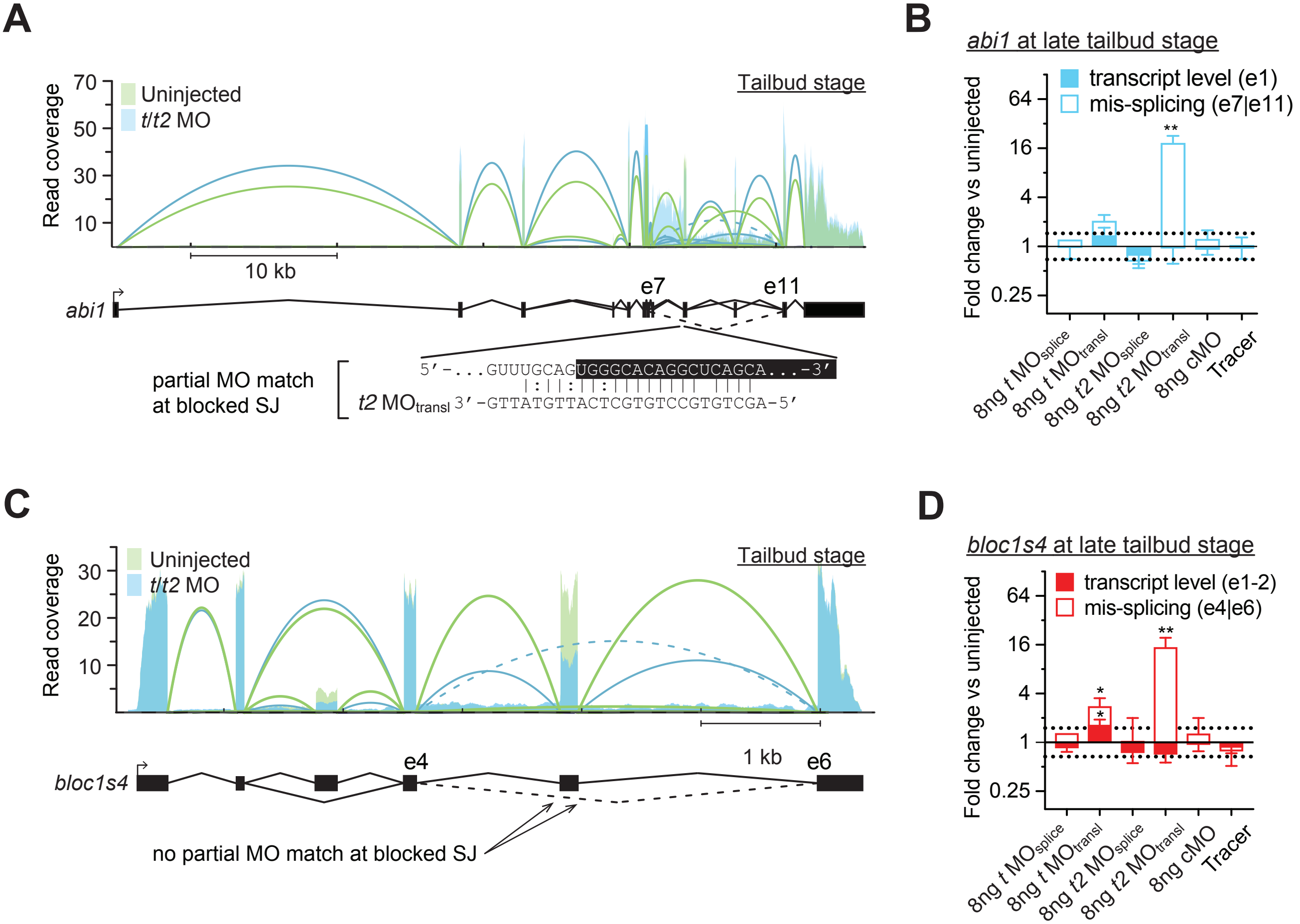
A
Read coverage
70
50
30
10
Uninjected
t/t2 MO
Tailbud stage
10 kb
abi1
e7
e11
partial MO match
at blocked SJ
5′-...GUUUGCAGUGGGCACAGGCUCAGCA...-3′
t2 MOtransl 3′-GTTATGTTACTCGTGTCCGTGTCGA-5′
B
abi1 at late tailbud stage
Fold change vs uninjected
64
16
4
1
0.25
transcript level (e1)
mis-splicing (e7|e11)
**
8ng t MOsplice
8ng t MOtransl
8ng t2 MOsplice
8ng t2 MOtransl
8ng cMO
Tracer
C
Read coverage
30
20
10
Uninjected
t/t2 MO
Tailbud stage
1 kb
bloc1s4
e4
e6
no partial MO match at blocked SJ
D
bloc1s4 at late tailbud stage
Fold change vs uninjected
64
16
4
1
0.25
transcript level (e1-2)
mis-splicing (e4|e6)
*
*
**
8ng t MOsplice
8ng t MOtransl
8ng t2 MOsplice
8ng t2 MOtransl
8ng cMO
Tracer

Figure S5

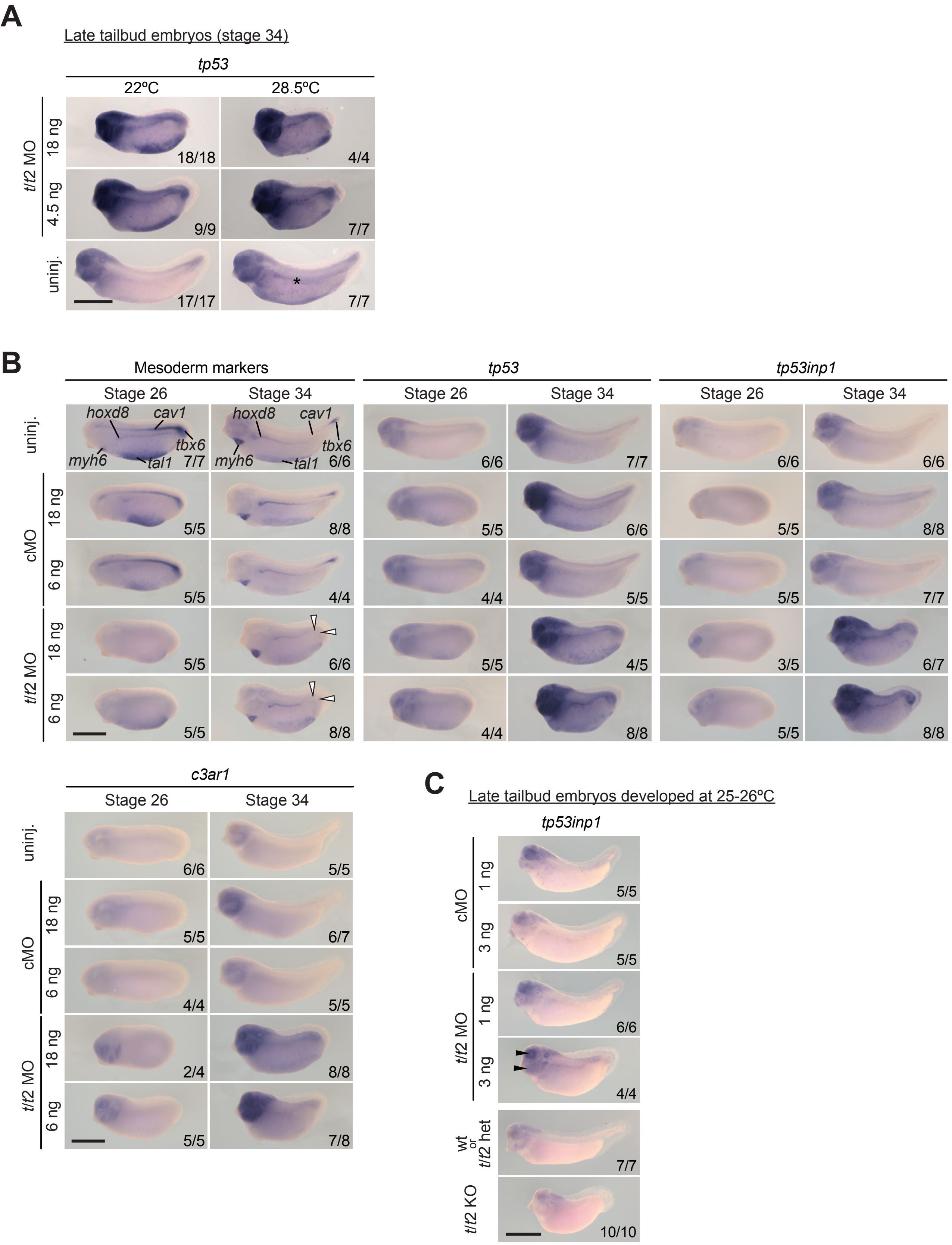
A
Late tailbud embryos (stage 34)
tp53
22ºC
28.5ºC
t/t2 MO
18 ng
4.5 ng
uninj.
18/18
4/4
9/9
7/7
17/17
7/7
B
Mesoderm markers
tp53
tp53inp1
Stage 26
Stage 34
hoxd8
cav1
tbx6
myh6
tal1
cMO
6 ng
c3ar1
C
Late tailbud embryos developed at 25-26ºC
1 ng
3 ng
wt or t/t2 het
t/t2 KO
5/5
6/6
4/4
10/10
