# Supplementary material for: Innate Immune Response and Off-Target Mis-splicing Are Common Morpholino-Induced Side Effects in Xenopus
Source: Dev Cell. 2018 Mar 12;44(5):597–610.e10. doi: 10.1016/j.devcel.2018.01.022 (PMC5861998; doi:10.1016/j.devcel.2018.01.022)
Supplement: Document S2. Article plus Supplemental Information [file mmc8.pdf]

Developmental Cell

Article

Innate Immune Response and Off-Target Mis-splicing Are Common Morpholino-Induced Side Effects in *Xenopus*

Graphical Abstract

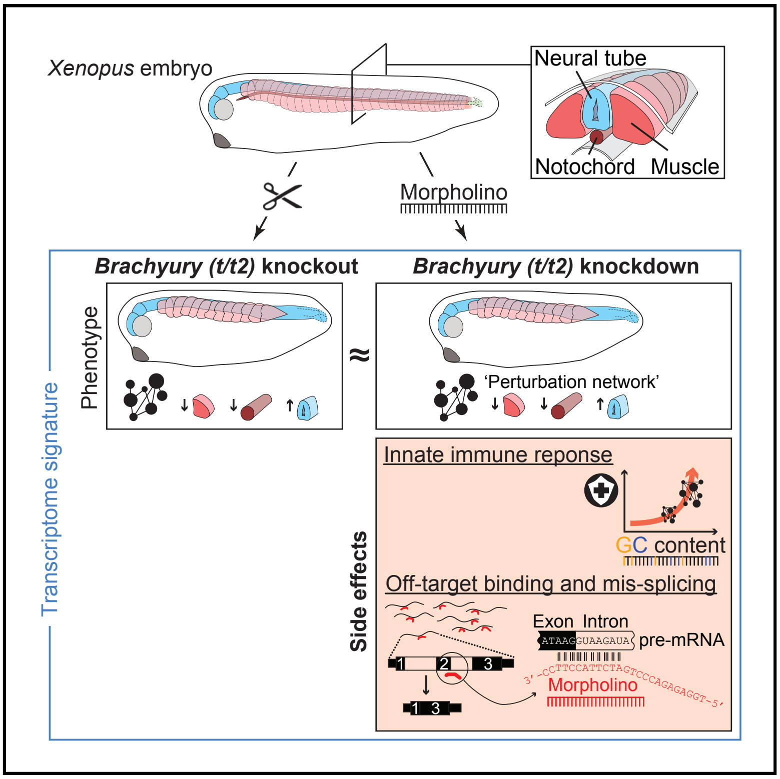

Authors

George E. Gentsch, Thomas Spruce, Rita S. Monteiro, Nick D.L. Owens, Stephen R. Martin, James C. Smith

Correspondence

george.gentsch@crick.ac.uk (G.E.G.), jim.smith@crick.ac.uk (J.C.S.)

In Brief

Antisense morpholino oligomers have frequently been used to knock down genes. By systematically comparing the knockdown and knockout of the same genes in frog embryos, Gentsch et al. reveal that morpholinos have significant side effects. They trigger an innate immune response and cause off-target splicing defects.

Highlights

- Brachyury KO and KD in frog equally affect phenotype-causing downstream genes
- Other transcriptional anomalies are unique to morpholino-based KDs and controls
- Morpholinos can trigger an innate immune response and off-target mis-splicing
- Optimization of KD conditions mitigates but does not eliminate these side effects

Gentsch et al., 2018, Developmental Cell *44*, 597–610
March 12, 2018 © 2018 The Francis Crick Institute. Published by Elsevier Inc.
https://doi.org/10.1016/j.devcel.2018.01.022

Developmental Cell
Article

Innate Immune Response and Off-Target Mis-splicing Are Common Morpholino-Induced Side Effects in *Xenopus*

George E. Gentsch,[1,5,*] Thomas Spruce,[1,3,5] Rita S. Monteiro,[1,5] Nick D.L. Owens,[1,4] Stephen R. Martin,[2] and James C. Smith[1,6,*]
[1]The Francis Crick Institute, Developmental Biology Laboratory, 1 Midland Road, London NW1 1AT, UK
[2]The Francis Crick Institute, Structural Biology Science Technology Platform, 1 Midland Road, London NW1 1AT, UK
[3]Present address: Centre for Genomic Regulation (CRG), The Barcelona Institute for Science and Technology, Dr. Aiguader 88, 08003 Barcelona, Spain
[4]Present address: Epigenetics of Stem Cells, Department of Stem Cell and Developmental Biology, Pasteur Institute, CNRS UMR3738, 25 Rue du Dr Roux, 75015 Paris, France
[5]These authors contributed equally
[6]Lead Contact
*Correspondence: george.gentsch@crick.ac.uk (G.E.G.), jim.smith@crick.ac.uk (J.C.S.)
https://doi.org/10.1016/j.devcel.2018.01.022

SUMMARY

Antisense morpholino oligomers (MOs) have been indispensable tools for developmental biologists to transiently knock down (KD) genes rather than to knock them out (KO). Here we report on the implications of genetic KO versus MO-mediated KD of the mesoderm-specifying *Brachyury* paralogs in the frog *Xenopus tropicalis*. While both KO and KD embryos fail to activate the same core gene regulatory network, resulting in virtually identical morphological defects, embryos injected with control or target MOs also show a systemic GC content-dependent immune response and many off-target splicing defects. Optimization of MO dosage and increasing incubation temperatures can mitigate, but not eliminate, these MO side effects, which are consistent with the high affinity measured between MO and off-target sequence *in vitro*. We conclude that while MOs can be useful to profile loss-of-function phenotypes at a molecular level, careful attention must be paid to their immunogenic and off-target side effects.

INTRODUCTION

Perturbing the function of a gene of interest in order to infer its biological role is a common approach in many biological disciplines including embryology and physiology. Since forward and reverse genetic approaches have not been readily applicable to many organisms, the injection of morpholino oligomers (MOs) has been widely adopted instead. This antisense technology is based on a nucleic acid analog with a backbone of morpholine rather than deoxyribose rings, and neutral phosphorodiamidate instead of negatively charged phosphate linkages. According to the manufacturer, MOs are more stable, efficient, and specific in knocking down genes than alternative knockdown (KD) technologies such as short interfering RNA, mainly because of their neutral features that prevent electrostatic interactions with endogenous proteins at physiological pH (Summerton, 2007). Depending on their design, MOs can block either translation or splicing when hybridizing almost perfectly over their full length of 23–25 bases to the translation start site or splice acceptor or donor site. In addition, they can disrupt the activity of non-coding RNA species, such as microRNAs (miRNAs) or Y-RNAs (Collart et al., 2011; Kloosterman et al., 2007).

While both anecdotal evidence and a number of published studies have suggested that MOs can cause off-target effects, it was previously assumed that, if appropriate control experiments are performed, robust knockout (KO)-like phenotypes could be generated. However, the use of TALEN and type II CRISPR genome editing technologies has now brought this assumption into question. Such studies have found that morphant and mutant phenotypes can be significantly different even when the morphant phenotype can be rescued by the introduction of cognate RNA species (Kok et al., 2015). While in some cases it is likely that the morphant phenotype is an off-target effect, in others it may be that the genetic mutation does not result in a complete loss of function either because it gives rise to a hypomorphic allele or because the gene product is maternally inherited (Blum et al., 2015). Mutating a gene can also in some circumstances lead to genetic compensation that is not triggered by MO-mediated KD (Rossi et al., 2015).

Nevertheless, bearing in mind that MOs can replicate corresponding null phenotypes at least at a morphological level, their use in vertebrate embryos may be legitimate and advantageous for several reasons. First, some maternal-effect genes are difficult to study due to an essential function in later life stages, and cumbersome germline-specific KO strategies are required to produce viable females with homozygous KO eggs (Liu et al., 2017). In particular, rapid-turnover proteins translated from maternal transcripts can be efficiently depleted with the injection of a translation-blocking MO into the zygote. In contrast, splice-blocking MOs will only perturb zygotic protein synthesis. Second, the simultaneous KD of multiple genes can reveal

This is an open access article under the CC BY license (http://creativecommons.org/licenses/by/4.0/).

functional redundancies (Gentsch et al., 2013; Khokha et al., 2005; Reversade et al., 2005), which can be informative for future KO strategies. However, this may require more MOs being injected into the embryo, thus increasing the likelihood of off-target effects. Third, MOs can be introduced into different mutant and transgenic backgrounds either to screen for genetic interactions or to help characterizing phenotypes, without the need for time-consuming intercrossing. Fourth, MO-injected embryos do not need genotyping, so that large numbers can be collected. This is of particular importance given the expanding use of molecular profiling to analyze loss-of-function effects at the chromatin level. However, this kind of differential analysis assumes that KD and KO animals with identical macro- or microscopic defects, such as in morphology or behavior, share similarly derailed genomic readouts. We examined this hypothesis in the western clawed frog *Xenopus tropicalis* by generating *Brachyury* null mutants using TALENs and comparing these with corresponding, previously validated morphants (Gentsch et al., 2013) at a transcriptome-wide level.

Our results showed that, while depletion of *Brachyury* resulted in the same dramatic loss of posterior mesoderm regardless of the gene interference technology employed, only control and *Brachyury*-targeting MOs perturbed hundreds of splicing events and caused excessive immune response-related gene transcription. These MO side effects were caused, on the one hand, by the off-target binding of premature transcripts and, on the other hand, by a cell-intrinsic (innate) immune reaction. The latter strongly correlated with the guanine-cytosine (GC) content of the injected MO. Proper dose and design optimization of the injected MO can mitigate these inadvertent effects. However, some specific off-target effects could not be eliminated even when an elevated incubation temperature was used in an effort to increase hybridization stringency. This is further corroborated by the kinetic analysis of MO oligomers binding off-target RNA sequences far below the minimal concentration required to produce a *t/t2* KO-like phenotype. We expect that our findings will be critical to keep unintended disruptions in tissue and organ development to a minimum.

RESULTS

TALEN-Induced Deletions Nullify Brachyury Function

In *Xenopus*, the function of *Brachyury* depends on two synexpressed and functionally redundant T-box transcription factors *t* (*Xbra*) and *t2* (*Xbra3*) (Gentsch et al., 2013; Hayata et al., 1999). We previously found that the combined injection of *t* and *t2* MOs produced a phenotype strongly resembling that of *Brachyury* null mice (Chesley, 1935; Gentsch et al., 2013). However, given recent controversies about MO specificity we sought to compare these *Brachyury* morphants with corresponding null mutants at a transcriptome-wide level in *X. tropicalis*. Two rounds of TALEN-induced mutagenesis were carried out in an effort to sequentially disrupt *t* and *t2* (Figure S1A). These paralogs are arranged in tandem on chromosome 5 within 30 kb and thus co-segregate during meiosis. First, *t* was mutated using a TALEN pair targeting the first SacI restriction site in exon 1 (Figure S1B). Animal or vegetal injection at the one-cell stage caused some disruption of the SacI site in ~90% of the embryos examined individually by PCR digest (animal 7/8, vegetal 9/10; Figure S1C). Sanger sequencing of PCR clones revealed indels of 1–6 base pairs (bp) (Figure S1D). About 80% of F_0 females raised to sexual maturity contained mutations in the germ line as confirmed by examining their offspring embryos. These embryos were used to generate lines of F_1 frogs with a variety of mutations in the *t* locus. In addition, homozygous offspring of F_0 *t* mutant intercrosses were short tailed, similar to previously published *t* morphants (Gentsch et al., 2013) (Figure S1E).

The second round of mutagenesis consisted of injecting F_2 heterozygous *t* mutant embryos with a TALEN pair targeting the only EcoRI restriction site in the third exon of *t2* (Figure S1F). Genotyping of injected embryos by PCR digest revealed ~30% (6/21) carried a mutation in the *t2* locus (Figure S1G). Tadpoles identified with mutations in *t2* were then raised to sexual maturity and three of the 15 frogs examined were found to have *t2*-specific germline mutations. Embryos from one of these frogs were found to have a 7-bp deletion in *t2* ($t2^{e3.7D}$) on the same chromosome as a 2-bp deletion in *t* ($t^{e1.2D}$). Both mutations were predicted to cause premature translation terminations before or midway through the critical T-box DNA binding domain by shifting stop codons into the reading frame (Figure 1A). However, these mutations did not cause any nonsense mediated decay of the transcript by neurula stage as revealed by qRT-PCR in $t^{e1.2D}$ and $t2^{e3.7D}$ hetero- and homozygotes (Figure 1B). In contrast, *t* transcript numbers increased 1.5- to 2-fold, indicating either increased stability of the mutant transcript or a fine-tuning of *t* transcription in response to a reduction or loss of functional Brachyury protein. The latter is similar to a previous observation reported for *vegfaa* mutants in zebrafish (Rossi et al., 2015). Since Brachyury directly regulates *t2* transcription (Gentsch et al., 2013), its complete loss led to a 5-fold reduction of *t2* expression during gastrulation (Figure 1B).

In order to confirm that $t^{e1.2D}$ and $t2^{e3.7D}$ contain null mutations, mRNAs encoding wild-type (WT) and mutant N- and C-terminally HA-tagged Brachyury were injected into *Xenopus* embryos (Figure S1H). We were unable to detect expression of the ~6 kDa product of N-terminally tagged $t^{e1.2D}$ by western blotting either because it is unstable or because of technical complications of blotting very short proteins. All other expected translation products were detected with no additional products being observed, indicating that neither *t* nor *t2* contain frequently used internal translational start sites. These mutant alleles lacked the ability of WT t and t2 to disrupt morphogenetic movements when expressed prematurely and ectopically (Figure S1I), so we conclude that these TALEN-induced deletions abolish *Brachyury* function.

Brachyury KO and KD Embryos Show Identical Mesoderm Defects

Crossing *X. tropicalis* frogs heterozygous for $t^{e1s.2D}$ and $t2^{e3.7D}$ (hereafter called t^- and $t2^-$) gave rise to the expected genotypes: WT ($t^{+/+}t2^{+/+}$), heterozygous (het, $t^{-/+}t2^{-/+}$), and homozygous (KO, $t^{-/-}t2^{-/-}$) embryos. Up to early tadpole stage 37, heterozygous embryos were indistinguishable under the stereo microscope from WT siblings or other WT embryos, including those injected with 18 ng control MO (cMO) (Figure S2A). By contrast, the combined disruption of both WT alleles of *t* and *t2* produced a consistent truncation of the embryonic tailbud and resulting tail, clearly visible by mid-tailbud stage 26 (Figure S2A). The

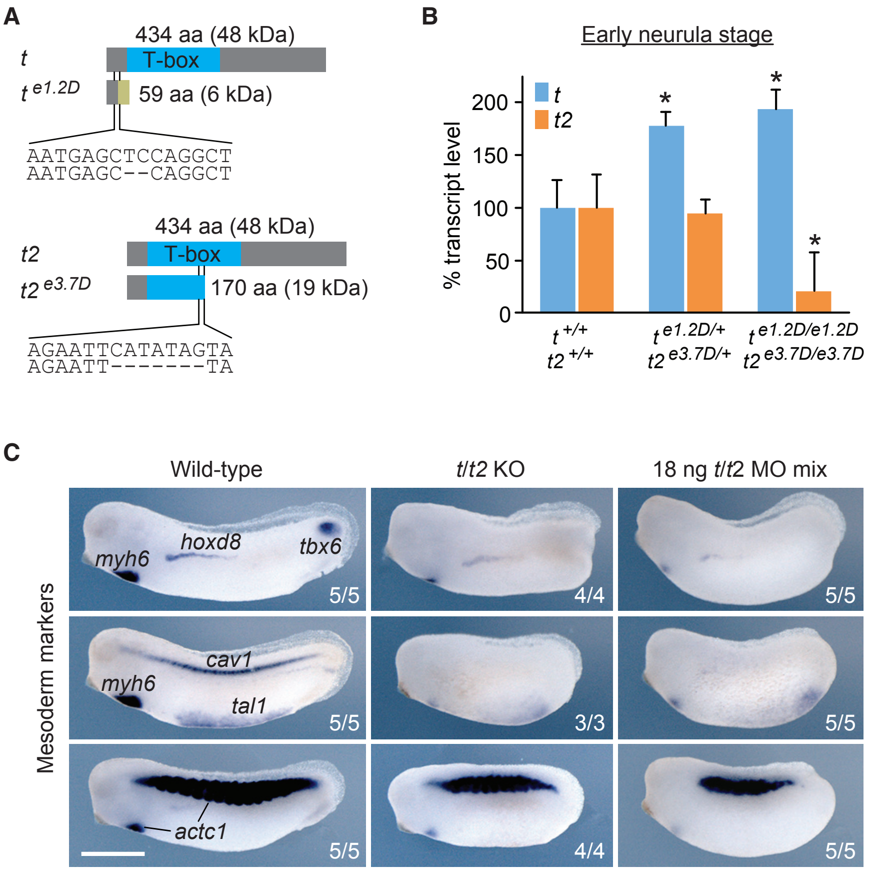

Figure 1. TALEN-Induced Deletions Nullify *Brachyury* Function

(A) TALEN-induced 2- and 7-bp deletions in exon 1 of *t* (e1.2D) and exon 3 of *t2* (e3.7D), and predicted frameshift translations generating truncated proteins of 59 and 170 amino acids (aa). These mutations were selected to generate a double heterozygous *X. tropicalis* line for the *Brachyury* paralogs *t* and *t2* ($t^{e1.2D/+}t2^{e3.7D/+}$).

(B) *t* and *t2* transcript levels in hetero- and homozygous embryos as measured by qRT-PCR at early neurula stage (n = 3, mean ± SD). Two-tailed t test: *p ≤ 0.05.

(C) Multi-probe WMISH for various mesoderm cell lineage and derivative markers (*actc1*; cardiac and skeletal muscle; *cav1*, notochord; *hoxd8*, pronephros; *myh6*, heart; *tal1*, ventral blood island; *tbx6*, paraxial mesoderm) in wild-type and Brachyury (*t/t2*) null (KO) embryos, as well as embryos injected with four MOs targeting *t* and *t2* (*t/t2* MO mix) at mid-tailbud stage. Scale bar, 0.5 mm.

morphology and timing of this developmental defect was virtually identical to that seen in embryos whose t and t2 protein levels were transiently depleted by the combined injection of four MOs (18 ng in total), one translation- and one splice-blocking MO (MO_{transl} and MO_{splice}) for each *Brachyury* gene (Figures S1B, S1F, and S2A). The efficiency of the MOs in blocking splicing or translation was previously verified by RT-PCR and western blotting (Gentsch et al., 2013). The intention of this combinatorial KD strategy were to increase KD efficiency and to mitigate side effects by reducing the dosage of individual MOs by using a pool of two MOs to target the same gene (Gentsch et al., 2013).

Multi-probe whole-mount *in situ* hybridization (WMISH) at mid-tailbud stage provided further evidence that genetic mutation and MO-mediated KD of *t* and *t2* similarly affect the spatiotemporal transcription of various mesodermal cell lineage and derivative markers (Figure 1C). Posterior mesoderm (*tbx6* and *T-box 6*) and its derivatives notochord (*cav1* and *caveolin 1*) and somites (*actc1* and *cardiac actin*) were absent or malformed, while the formation of anterior, intermediate, and ventral mesoderm subtypes such as heart (*myh6* and *myosin heavy chain 6*), pronephros (*hoxd8*), and blood (*tal1* and *T cell acute lymphocytic leukemia 1*) was initiated, albeit with some delay (see also Figures 7B, 7E, and S5B).

Morpholinos Can Trigger an Immune Response

Since recent studies in zebrafish claim low concordance between mutant and morphant phenotypes (Kok et al., 2015), we sought to compare the entire poly(A) transcriptome over two tailbud stages (mid-tailbud stage 26 and late tailbud stage 34 separated by ~12 hr of development at 25°C) using deep RNA sequencing (RNA-seq) (Table S1) and likelihood ratio tests (Table S2) (Love et al., 2014). Biological triplicates were used to account for transcriptional variability between clutches. Libraries were generated simultaneously to mitigate any batch effects. Most of the transcriptional changes observed were attributed to the developmental stage (principal component 1 [PC1]: ~65%) and to the treatment and genotype (PC2: ~14%) of the different samples: uninjected, control, and *t/t2* MO-injected embryos from three independent KD experiments; and WT, heterozygous and homozygous embryos from three separate crosses between $t^{-/+}t2^{-/+}$ heterozygotes (KO experiment; Figure 2A). As expected from gross comparison of morphology and mesoderm markers developmental stage-equivalent WT, heterozygous (*t/t2* het), and uninjected embryos (unlabeled in Figure 2A) shared a very similar transcriptome. However, the transcriptome of control (cMO) and *t/t2* (*t/t2* MO) morphants deviated considerably from their genetic counterparts, and more so at the later stage (Figure 2A).

A pairwise comparison of the transcript levels of 17,716 genes (showing ≥7 fragments among "control" conditions; Figure S2B) between the different conditions revealed that control and *t/t2* morphants had significantly more mis-regulated mRNA (≥1.5-fold change at false discovery rate [FDR] ≤10%) than *t/t2* hetero- and homozygous embryos (1.61% versus 0.05% and 14.85% versus 3.53%), respectively (Figure 2B). Thus, in a Venn diagram the groups of down- and upregulated genes unique to *t/t2* MO (Venn field C) were ~3- and ~38-fold larger, respectively, than the overlap between *t/t2* MO and KO embryos (field D) (Figure 2C; Table S3). Three other Venn fields also contained a significant number of genes: the overlap between cMO and *t/t2* MO (field B) and the fields unique to cMO (field A) and *t/t2* KO (field E). The remaining Venn fields contained no or only a few genes and were excluded from further analysis.

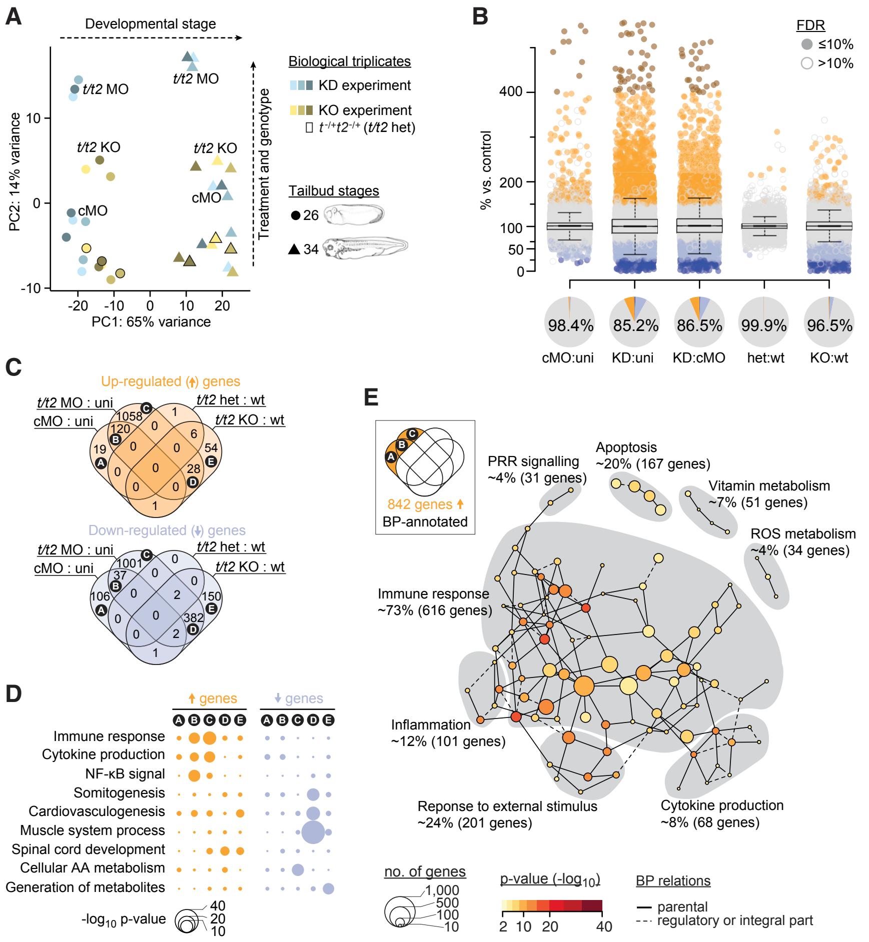

Figure 2. Transcriptional Deviation from Genetic Counterpart Reveals Immune Response as MO Side Effect

(A) Principal component (PC) analysis of poly(A) RNA profiles at indicated tailbud stages resulting from biological triplicates of a *t/t2* KD and KO experiment. The KD experiment involved uninjected (not labeled), control (cMO), and *t/t2* MO-injected embryos. The KO experiment consisted of wild-type (WT) (not labeled), heterozygous ($t^{-/+}t2^{-/+}$; data points framed in black, not labeled) and homozygous (*t/t2* KO) embryos.

(B) Jitter/boxplot and pie chart show pairwise transcriptional comparisons of cMO, *t/t2* MO (KD), heterozygous (het), KO embryos with uninjected (uni), cMO, or wild-type (WT) embryos. Only fold changes with FDR ≤10% were colored: navy blue <25%, sky blue 25%–67%, orange 150%–400%, and red >400% compared with reference transcript level. Percentage number in pie chart represents percentage of genes that were unaffected (i.e., whose fold change are <1.5 or FDR >10% between indicated conditions).

(C) Venn diagram of genes with increased and decreased transcript levels (i.e., ≥1.5-fold change at FDR ≤10%). See Table S3 for corresponding gene list.

(D) Statistical significance (hypergeometric p value) of enrichment for some selected biological processes (BPs) among the indicated Venn fields.

(E) MO-triggered transcriptional signature of an immune response. Gray areas represent Newman-Girvan-based communities of enriched BPs associated with 842 genes in fields A, B, and C of the Venn diagram. See Table S4 for corresponding and other Venn field-specific gene set enrichment analyses.

Functional "perturbation networks" were then derived from the biological processes (BPs) of the gene ontology (GO) project that were significantly ($p \leq 0.0001$) enriched in the five largest Venn fields and divided into GO-linked Newman-Girvan (NG) communities (Table S4). As expected from a vertebrate *Brachyury* phenotype, ~80% (256 genes) of the downregulated BP-annotated (Table S4) genes shared between KD and KO embryos were associated with the development, the anatomy, and the physiology of mesoderm and its derivatives muscle and heart, such as the formation of somites and muscle fibers, the contraction of tissue, and calcium homeostasis (Figure 2D; Table S4). In addition, >70% of the upregulated BP-annotated genes either shared between KD and KO embryos or unique to the latter were enriched for neural development, including the process of neurotransmission and spinal cord formation (Figure 2D; Table S4). The low number of NG communities in both perturbation networks required to contain most mis-regulated genes suggested the predominant role of Brachyury in regulating the neuromesodermal cell trajectory (Table S4). Overall, these genes represented the *Brachyury* phenotype-defining core regulatory network. However, the largest group of genes with a uniform BP signature were upregulated only in control (cMO) and/or *Brachyury* (*t/t2* MO) morphants (Figures 2C–2E and Table S4): ~650 of these were characteristic of an immune response containing pro-inflammatory mediators, and components of the pattern recognition receptor (PRR) and nuclear factor κB (NF-κB) signaling pathways (Figures 2D and 2E; Table S4). The wide-spread mis-regulation of immune response related genes such as Toll-like receptors (TLRs), complement components, cytokines, caspases, and tumor suppressors was a dramatic side effect of injecting control or *t/t2* MOs, since their induction or suppression was unique to morphants, and their local chromatin environment in early tailbud embryos did not show any chromatin occupancy of Brachyury (t) as detected by chromatin immunoprecipitation sequencing (Gentsch et al., 2013) in contrast to the Brachyury-dependent core regulatory network (Figure 3A).

Spatiotemporal expression profiling of the *complement component 3a receptor 1* (*c3ar1*), *tumor protein 53* (*tp53*), and *tp53 inducible nuclear protein* (*tp53inp1*) indicated that the immune response was ubiquitous and intensified beyond post-neurula stages (Figures 3B–3D). Higher off-target transcript levels were detected in WT expression domains such as the eye, the branchial arches, and the nervous and cardiovascular systems. As in some zebrafish morphants and various tumors (Bourdon et al., 2005; Khoury and Bourdon, 2010; Robu et al., 2007), the elevated *tp53* expression level was largely driven from an internal promoter ~150 bp upstream of exon 5 (Figure 3D). This produces an N-terminally truncated tp53 isoform specifically containing 20 amino acids encoded by the acceptor

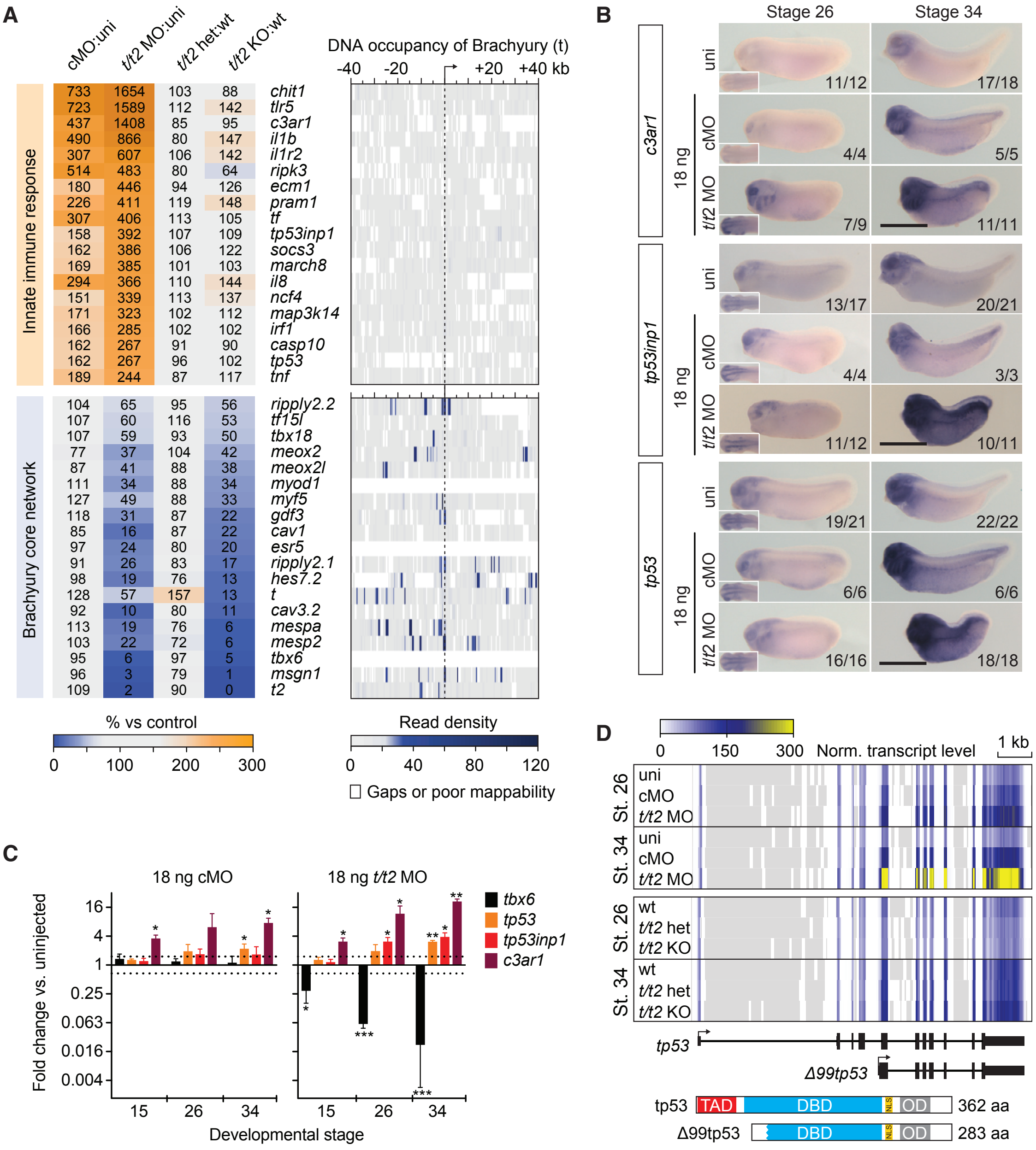

Figure 3. Ubiquitous Immune Response against MO Intensifies during Embryogenesis

(A) Panel of genes upregulated in control and *t/t2* morphants associated with the immune response and genes downregulated in *t/t2* morphants and null mutants representing the Brachyury-dependent core network. Heatmap to the right represents the binding map of Brachyury (t) in the proximity (±40 kb) of indicated transcription start sites (TSS) at early tailbud stage (Gentsch et al., 2013).

(B) WMISH of immune response related gene transcripts *c3ar1*, *tp53inp1*, and *tp53* in uninjected (uni) embryos and embryos injected with 18 ng of cMO or *t/t2* MO mix. Left bottom corner inset, dorsal view of tailbud head showing elevated transcript levels in the CNS. *tp53* antisense probe did not discriminate active isoforms shown in D. Scale bar, 0.5 mm.

(C) Temporal dynamics of transcript fold changes (log_2 scale) for a selected group of genes representing the Brachyury-directed core network (*tbx6*) and the immune response (*c3ar1*, *tp53inp1*, and *tp53*) in MO-injected versus uninjected embryos as measured by qRT-PCR (n = 3, mean ± SD). Two-tailed t test ($\geq$1.5-fold change): *p $\leq$ 0.1; **p $\leq$ 0.01; and ***p $\leq$ 0.001.

(D) Genome map of full length *tp53* and *Δ99tp53* transcript isoforms shows normalized transcript levels for uninjected (uni), control morphants (cMO), *t/t2* morphants (*t/t2* MO), wild-type (WT), *t/t2* heterozygous (*t/t2* het), and homozygous (*t/t2* KO) mutant embryos at tailbud stages 26 and 34. Isoform-corresponding translation products with critical domains are on display below the heatmap: TAD, transactivation domain; DBD, DNA binding domain; NLS, nuclear localization signal; and OD, oligomerization domain.

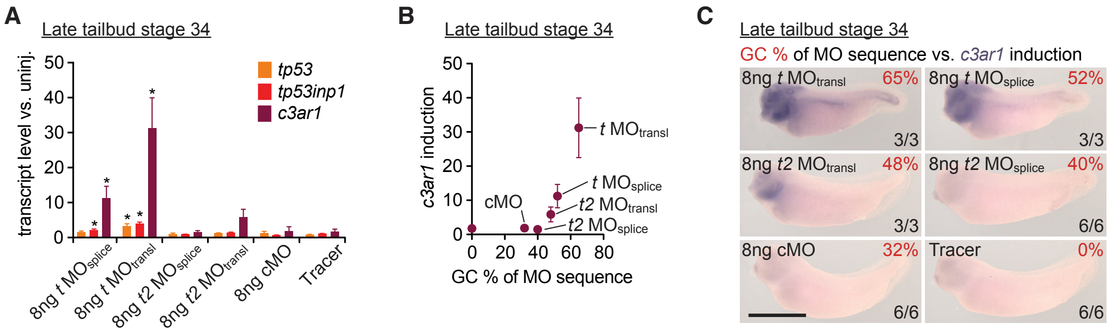

Figure 4. Intensity of Innate Immune Response Depends on GC Content of MO

(A) Assigning the induction of immune response-related genes *tp53*, *tp53inp1*, and *c3ar1* to single splice- or translation-blocking MOs (MO_{splice} and MO_{transl}) of the *t/t2* MO mix injected at 8 ng per embryo as well as to the cell lineage tracer sulforhodamine (coupled to dextran). The transcript fold change was determined at late tailbud stage by qRT-PCR (n = 4, mean ± SD). Two-tailed t test: *p ≤ 0.1.

(B and C) *c3ar1* induction was increasing with the GC content of the injected MO as determined by qRT-PCR (n = 4, mean ± SD) and WMISH. Scale bar, 0.5 mm.

splice arm of intron 4 instead of the 99 amino acids encoded by exons 2 to 4 (Δ99tp53). Thus, Δ99tp53 lacks the pro-apoptotic p53 transactivation domain, but retains most of the DNA binding domain (except for the first 31 amino acids), its full nuclear localization signal, and oligomerization domain (Figure 3D). Similarly truncated tp53 isoforms, such as Δ113tp53 in zebrafish and Δ133tp53 in human, have been reported to suppress apoptosis by lowering transcriptional activity mediated by full-length tp53 (Bourdon et al., 2005; Chen et al., 2009). Consistent with the presumed anti-apoptotic function of elevated Δ99tp53, the MO-mediated KD did not cause more apoptosis at the late tailbud stage than was observed in the *t/t2* KO or any control condition as detected by terminal deoxynucleotidyl transferase dUTP nick end labeling (TUNEL) (Figure S3A).

In order to resolve the immune response into single MOs, including any fluorescent tag used for cell tracing, all MOs and the tracer sulforhodamine coupled to dextran were injected separately and compared with uninjected embryos (Figures 4A–4C and S3B). At a dose of 8 ng per embryo, which is 10 ng less than the dose used for *t/t2* MO mix or cMO in the original KD experiment, MOs with a GC content above 40% showed an elevated immune response as judged by the gene induction of *tp53*, *tp53inp1*, and *c3ar1* in late tailbud embryos. The *t* MO_{transl} with the highest GC content (65%) among all MOs used in this study triggered the strongest response followed by *t* MO_{splice} (52%) and *t2* MO_{transl} (48%). A dose of 8 ng of *t2* MO_{splice} (40%) and cMO (32%), as well as the tracer on its own, did not show any significant immunogenic properties in *X. tropicalis* embryos with respect to the induction of these genes.

Morpholinos Can Cause Off-Target Mis-splicing

The *Brachyury* phenotype-related and immune response BP signatures we observed were fairly robust. However, with the exception of some metabolic anomalies, which may provide orthogonal information (Mülleder et al., 2016) about the effects of transient or permanent loss of Brachyury protein (Figure 2D; Table S4), other genes with decreased transcript levels that were detected uniquely in *t/t2* null or in morphant embryos showed only weak and heterogeneous BP associations.

The large number of "mis-regulated" genes in morphants that lacked any significant BP communities prompted us to look into MO-specific off-target effects such as mis-splicing, which might affect transcript stability (Lykke-Andersen and Jensen, 2015). In contrast to MO-mediated inhibition of translation, a splicing defect should be readily detectable by the computational analysis of sequencing reads across at least two splice junctions (see d and cr_{1-4} in Figure 5A). The hybridization of an MO to a pre-mRNA splice site, such as the target donor site (d) of intron 1 of the *t* transcript, decreases canonical splicing while forcing intron retention or (partial) exon skipping due to increased alternative or cryptic splicing (cr_1-cr_4). Since the splice blocking MOs for *t* and *t2*, as well as the standard cMO from Gene Tools (cMO) contained at least seven consecutive bases perfectly matching the most frequently encountered canonical donor splice site in *Xenopus* (Figure 5B) and other vertebrate species, it seemed plausible that hybridization and interference with splicing could occur in an off-target manner. cMO was originally designed to block an aberrant donor splice site formed by the single-point (705T > G) mutation in the second intron of human β-globin that causes a blood disorder known as β-thalassemia (Kang et al., 1998).

Using an annotation-free quantification method, 758 and 153 intron clusters were found (FDR ≤ 0.001%) to be differentially spliced in *t/t2* and control morphant embryos, respectively, compared with all other conditions including *t/t2* null embryos (Figure 5C; Table S5). Introns were clustered according to the common usage of splice sites (e.g., acceptor [a] site in Figure 5A). As expected, differential intron usage within a cluster showed increased alternative (alt) or cryptic (cr) splicing (Figure 5C and Table S5) at the expense of canonical (can) splicing with some clusters being affected by both *t/t2* MOs and cMO. The blocked splicing sites showed more consecutive base pairing with the injected MOs (Mann-Whitney U p value $<2.2 \times 10^{-16}$ for cMO and 7.8×10^{-15} for *t/t2* MOs; see legend for expected and observed numbers) and were more frequently associated with decreased transcript levels than randomly selected active splice sites (Mann-Whitney U p value $<2.2 \times 10^{-16}$ for both cMO and *t/t2* MOs). Mis-spliced genes were not enriched for any GO term, suggesting that off-target MO hybridization was not preferentially affecting any biological function (data not shown). Four of these MO-enhanced splicing events, including the MO target gene *t*, were confirmed by qRT-PCR (Figure 5D) from mid-neurula to

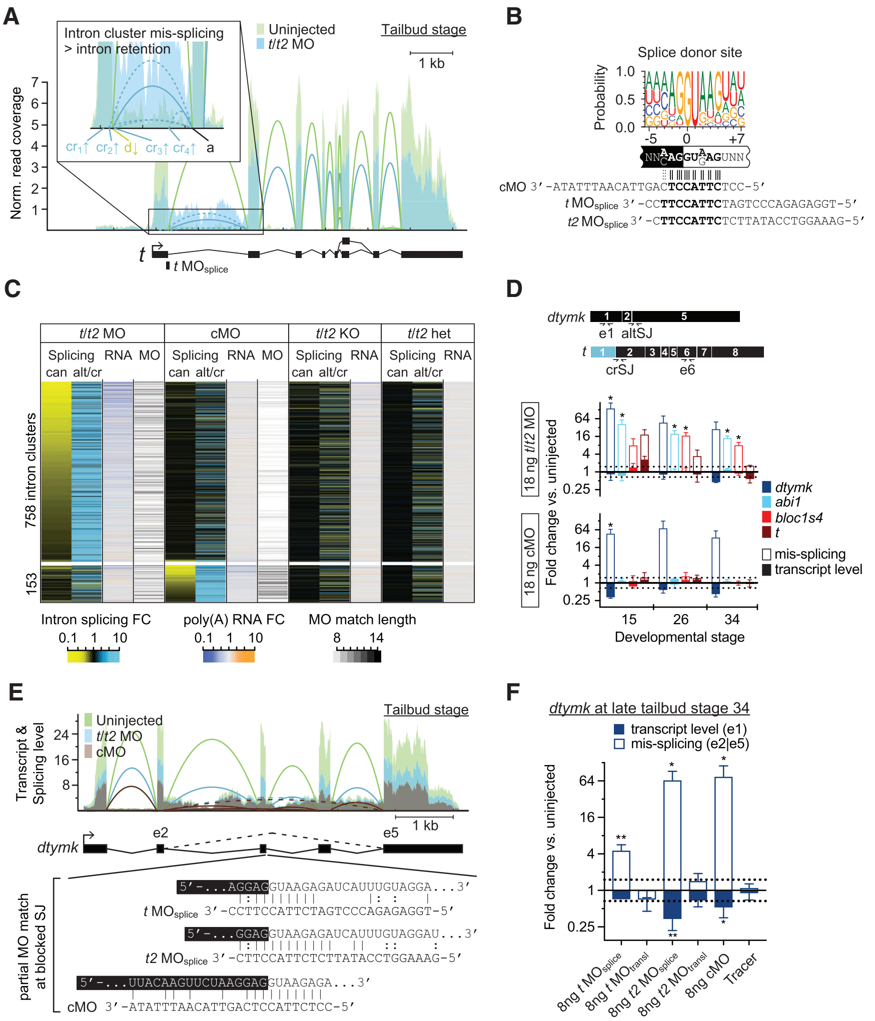

Figure 5. MOs Can Cause Off-Target Mis-splicing

(A) Quantitative comparison of *t* transcript levels and splice junction usage between *t/t2* MO-injected and uninjected tailbud embryos in a superimposed Sashimi plot. Canonical and cryptic splicing are shown with solid and dashed lines, respectively. Magnification of the first intron indicates position and altered usage (see arrow after abbreviation for various splice sites) of splice junctions caused by the *t* donor splice-blocking MO (MO_{splice}): a, acceptor splice site; d, canonical donor splice site; cr_{1-4}, cryptic splice sites.

(B) Consensus sequence of all canonical donor splice junctions detected in the transcriptome of *X. tropicalis* tailbud embryos and best alignment with control and donor splice-blocking MOs.

(C) Seriated heatmap of differentially used intron clusters, transcript level changes, and MO match length at blocked splice junctions for indicated conditions compared with their uninjected controls. Selected intron clusters represent coupled splicing events that were inversely mis-regulated in either *t/t2* MO- or cMO-injected tailbud embryos: inhibition of canonical (can) splicing caused alternative or cryptic (alt/cr) splice sites to be used more frequently. Blocked splice sites, 758 in *t/t2* morphants and 153 control morphants, were observed with higher occurrences of reduced transcript levels (<67%; 165:54 and 19:1, respectively) and more consecutive MO base pairing (≥10; 92:26 and 49:2, respectively) than expected (n_{obs}:n_{exp}).

(D) Temporal dynamics of mis-splicing (solid bar) and transcript (filled bar) fold changes (log_2 scale) for transcripts *dtymk*, *abi1*, *bloc1s4*, and *t* in control (cMO) and *t/t2* morphants (*t/t2* MO) from neurula (stage 15) to mid-tailbud (stage 26) to late tailbud stage (stage 34). Mis-splicing was quantified by qRT-PCR (n = 3, mean ± SD) using forward primers that span alternative or cryptic splice junctions (altSJ/crSJ) as shown above the bar graph for *dtymk* and *t*. Cryptic splice junction shortens first exon of *t* (colored blue). Changes in transcript levels were determined at indicated exons (e). See Key Resources Table for the design of qRT-PCR primers.

(E) Superimposed Sashimi plot of transcript *dtymk* whose splicing was affected by both cMO and *t/t2* MO at tailbud stage. Canonical and alternative (between exons 2 and 5) splicing are shown with solid and dashed lines, respectively. The blocked donor splice site featured partial matches of ≥8 consecutive bases with the MO_{splice} of *t* and *t2* as well as the cMO. The alignments show canonical Watson-Crick (vertical bar) and non-canonical wobble (colon) base pairing between the transcript and several MOs.

(F) Confirmation of the alignment-based predictions in E by injecting single MOs or tracer sulforhodamine as indicated. Fold changes (log_2 scale) to the alternative splicing and transcript level of *dtymk* were quantified by qRT-PCR (n = 4, mean ± SD).

Two-tailed t test: *p < 0.1; **p < 0.01.

late tailbud stage using a forward primer that spanned an alternative or cryptic splice junction (altSJ or crSJ). cMO and *t/t2* MOs caused a 10–100× increase in *dtymk* transcripts without exons 3 and 4, while the whole transcript level measured at exon 1 (e1) dropped slightly (Figure 5D). cMO and *t/t2* MO_{splice} matched the blocked donor splice site of intron 3 of *dtymk* at ≥8 consecutive Watson-Crick base pairs (Figure 5E). Since wobble interactions between guanine and thymine or uracil show similar thermodynamic stabilities and superior conformational flexibilities to Watson-Crick base pairing (Varani and McClain, 2000), this putative hybridization length could increase to ≥10 (Figure 5E). Other transcripts showing *t/t2* MO-induced alternative splicing were *abi1* and *bloc1s4*. However, their transcript levels were little affected by MO injection (Figures 5D, S4A, and S4C). Intron 8 of *abi1* was retained in *t/t2* morphants, which coincided with a 21-bp alignment between *t2* MO_{transl} and the correspondent splice acceptor site containing 1 mismatch and 3 wobble positions (Figure S4A). The blocked spliced sites of the gene *bloc1s4* did not show any perfect alignment of ≥8 bp to any of the *t/t2* MOs (Figure S4C). However, as with many other detected mis-splicing events other intronic or exonic *cis*-regulatory splicing motifs more distant from the affected splice site might be blocked by off-target MO hybridization.

To corroborate some of our potential off-target sites, mis-splicing was quantified in tailbud embryos injected with single splice- or translation-blocking MOs, cMO, or the tracer moiety sulforhodamine coupled to dextran (Figures 5F, S4B, and S4D). As predicted, only MOs with partial matches at the blocked splice junctions of *dtymk* and *abi1* induced alternative splicing. However, predictions can be difficult, as shown by the gene

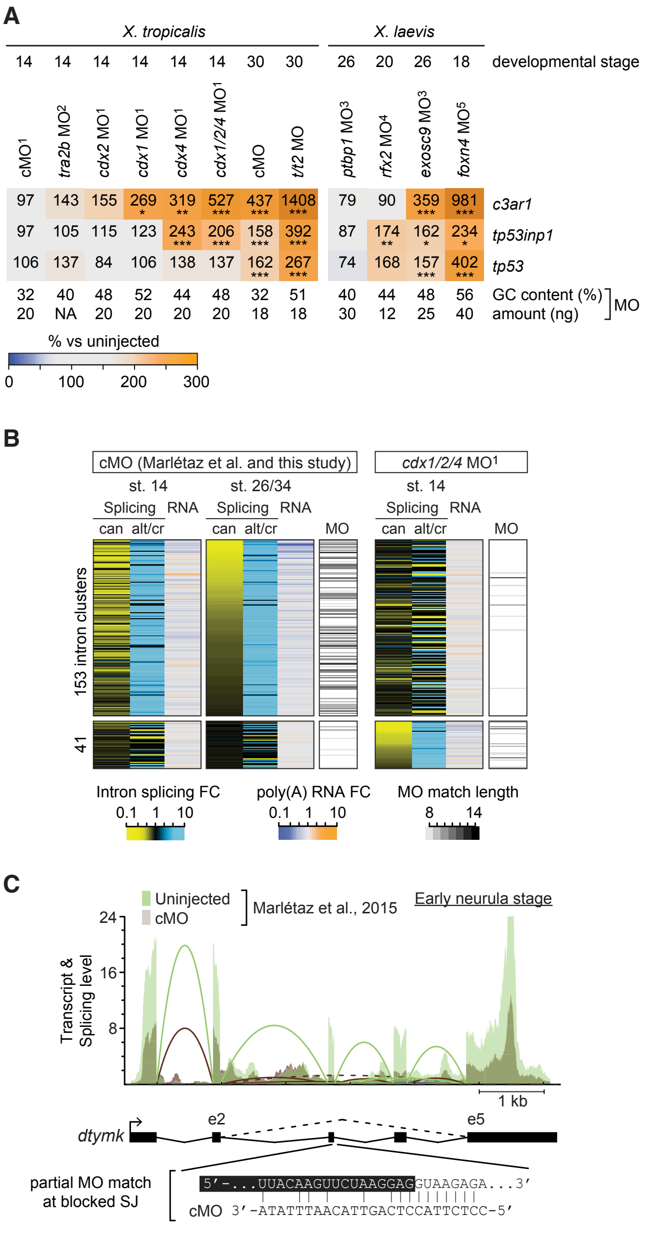

Figure 6. Analysis of Public RNA-Seq Datasets Substantiates Identified MO Side Effects

Superscripts refer to the published datasets used in this study: [1]Marlétaz et al., 2015; [2]Dichmann et al., 2015; [3]Noiret et al., 2016; [4]Chung et al., 2014; and [5]Campbell et al., 2016.

(A) Heatmap of MO-induced transcriptional mis-regulation (%, percentage compared with uninjected embryos) of *c3ar1* (*c3ar1.L*), *tp53inp1* (*tp53inp1.L*), and *tp53* (*tp53.L*) in *X. tropicalis* and *X. laevis* (gene names in brackets) embryos at indicated developmental stages. Asterisks indicate statistical significance: *FDR $\leq$10%; **FDR $\leq$1%; and ***FDR $\leq$0.1%. The (average) GC content and dosage of MO(s) are listed below the heatmap.

(B) Seriated heatmap of splice/transcript levels and MO match length at blocked splice junctions for MO-injected embryos compared with their uninjected controls. Selected intron clusters represent coupled splicing events that were inversely mis-regulated in embryos injected with cMO (153 intron clusters at tailbud stage) or the *cdx1/2/4* MO mix (41 intron clusters at neurula stage): inhibition of canonical (can) splicing caused alternative or cryptic (alt/cr) splice sites to be used more frequently. Intron cluster-specific heatmap rows were sorted by the mis-regulation of canonical splicing in morphants.

(C) Superimposed Sashimi plot of transcript *dtymk* whose splicing was affected by cMO at early neurula stage (data from Marlétaz et al., 2015). Canonical and alternative (between exons 2 and 5) splicing are shown with solid and dashed lines, respectively. The blocked donor splice site of intron 3 contains 10 consecutive bases perfectly complementary to sequence of the cMO.

example *bloc1s4*, which displayed alternative splicing in the presence of *t2* MO_{transl} without any obvious partial matches in close proximity of the blocked splice junction.

Optimized Conditions Do Not Eliminate Morpholino Side Effects

Our re-analysis of public RNA-seq datasets (Campbell et al., 2016; Chung et al., 2014; Dichmann et al., 2015; Marlétaz et al., 2015; Noiret et al., 2016)—with the caveat that KO references were not available for these—reinforced that the immune response and mis-splicing were likely common side effects of MO-mediated KDs in *Xenopus* embryos (Figure 6). The strength of immune response as inferred from the transcriptional induction of *c3ar1*, *tp53inp1*, and *tp53* increased with developmental progression as well as the GC content and the amount of the injected MO (Figure 6A). The comparison of mis-splicing defects induced by the cMO between early neurula (Marlétaz et al., 2015) and tailbud (this study) embryos confirmed the strong correlation with cMO-complementary sequence at blocked splice junctions (e.g., donor splice site of intron 3 of *dtymk*) and suggested that these off-target effects accumulate over time as more genes are transcribed (Figures 6B and 6C).

Various approaches have been either suggested or used to mitigate MO side effects, including co-injecting *tp53* MO to attenuate apoptosis or increasing incubation temperature to reduce off-target MO hybridization (Eisen and Smith, 2008; Robu et al., 2007). We refrained from interfering with *tp53* because of its essential role in stabilizing the genome (Khoury and Bourdon, 2010; Lane, 1992). Here, we increased the incubation temperature or reduced the MO dose or both in an effort to minimize MO side effects while retaining the *Brachyury* phenotype (Figure 7). Increasing the incubation temperature from 22°C to 28.5°C did not significantly reduce the transcriptional immune response (*tp53*, *tp53inp1*, and *c3ar1*) or off-target mis-splicing (*dtymk*, *abi1*, and *bloc1s4*) in *t/t2* morphants irrespective of the dose of 4.5 or 18 ng of the *t/t2* MO mix per embryo (Figures 7A–7C and S5A). Nevertheless, higher incubation temperatures could have some overall effects on MO stringency, as the lower dose of 4.5 ng of the *t/t2* MO mix per embryo was more efficient at blocking Brachyury-dependent genes at 28.5°C than at 22°C (see white arrowheads in Figure 7B). It is plausible that less off-target hybridization at higher temperatures made more MO oligomers available for the *Brachyury* KD. The 4-fold reduction of the MO dose slightly reduced mis-splicing and recovered some of the transcript loss of *dtymk* and *bloc1s4* (Figure 7C). Yet, it did not lower excessive gene activation of *tp53*, *tp53inp1*, and *c3ar1* (Figure 7A).

Finally, while 10–20 ng of MOs is frequently used in *X. tropicalis* to elicit gene KD (Bogdanović et al., 2016; Gentsch et al., 2013; Nakamura et al., 2016; Yasuoka et al., 2014), we found by serial

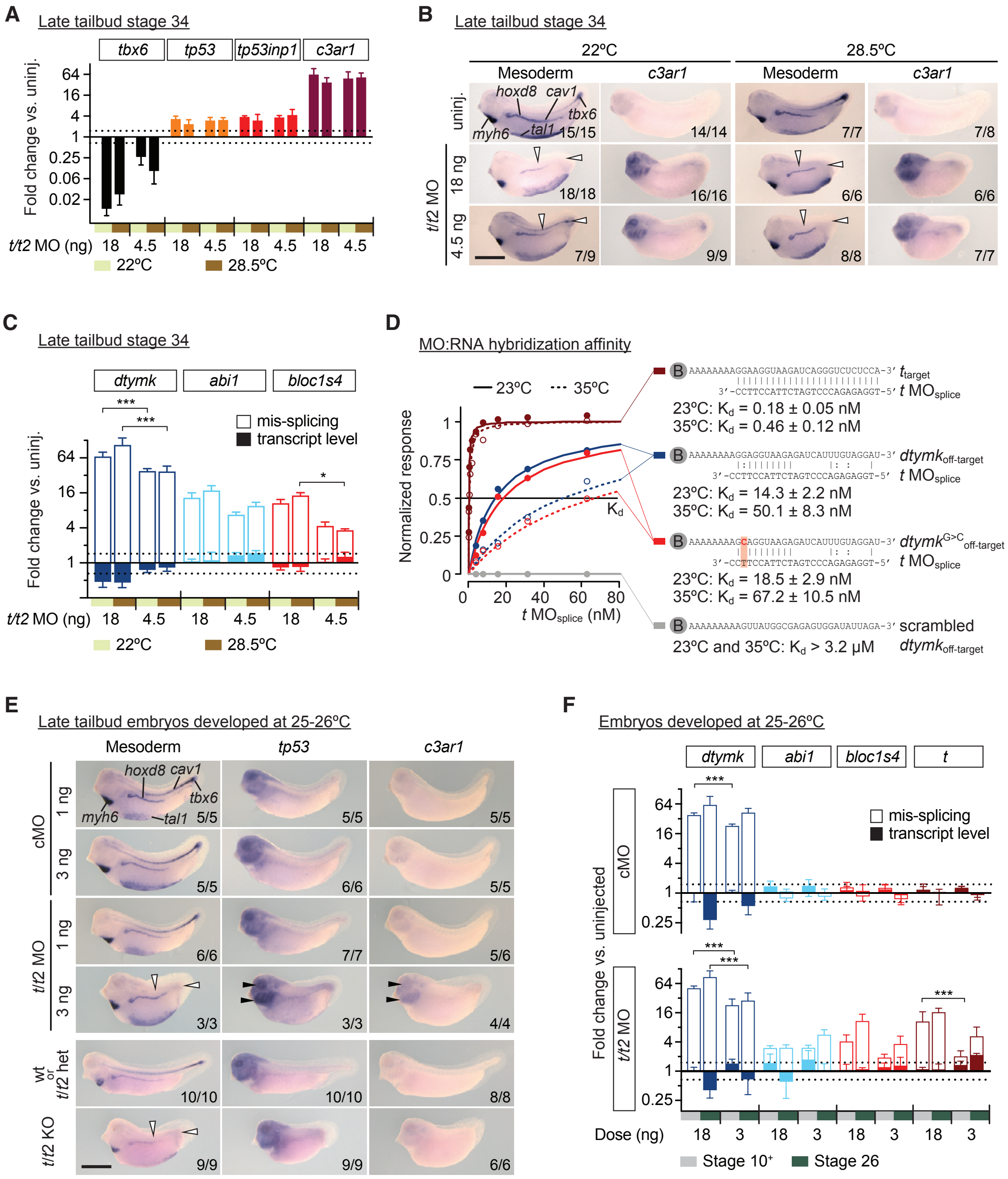

Figure 7. Optimizations of KD Conditions Can Reduce but Fail to Eliminate MO Side Effects

(A–C) Fold changes to transcript levels and alternative splicing under different doses of the *t/t2* MO mix (4.5 or 18 ng) and incubation temperatures (22°C or 28.5°C) were quantified at late tailbud stage by qRT-PCR (n = 4, mean ± SD) or WMISH. Two-way ANOVA test: $*p < 0.1$; $***p < 0.001$. (A) Transcriptional mis-regulation (log_2 scale) of the Brachyury target gene *tbx6* and of MO side effect genes *tp53*, *tp53inp1*, and *c3ar1*. (B) Multi-probe WMISH for various mesoderm cell lineage and derivative markers (*cav1*, notochord; *hoxd8*, pronephros; *myh6*, heart; *tal1*, ventral blood island; *tbx6*, paraxial mesoderm) and single WMISH for the immune response-related gene *c3ar1*. White arrowheads point to the expression domains of *tbx6* and *cav1* that were not maintained in embryos without functional Brachyury. (C) Fold changes (log_2 scale) to the alternative splicing (solid bar) and transcript level (filled bar) of the "off-target" genes *dtymk*, *abi1*, and *bloc1s4*.

(D) Measurements of the hybridization affinity (equilibrium dissociation constant K_d) between t_{splice} MO and target or off-target (including a point-mutant and a scrambled version) RNA oligonucleotides at 23°C and 35°C using biolayer interferometry (normalized instrument response versus MO concentration). The off-target sequence was derived from the most likely blocked site causing *dtymk* mis-splicing (see Figure 5E).

(legend continued on next page)

dilution that the effective dose of the *t/t2* MO mix could be reduced by about 6-fold to ~3 ng per embryo (i.e., ~750 pg per MO) at 25°C to 26°C (Figures 7E, S5B, and S5C). This considerably reduced excessive activation of *c3ar1*, *tp53*, and *tp53inp1*, although some overexpression could still be detected in the eye and the pharyngeal arches (see black arrowheads in Figure 7E). However, this low MO dose still failed to strongly mitigate aberrant splicing detected as early as the onset of gastrulation (Figure 7F). Moreover, the reduction of the *t/t2* MO dose lowered mis-splicing equally in both target *t* transcripts and in off-target transcripts such as *dtymk*, *abi1*, and *bloc1s4* (Figure 7F). Thus, decreasing MO dose to a level that still retained the phenotype was able to reduce, but not abolish, MO-mediated immune response or off-target splicing defects.

While the immune response could be further moderated by redesigning MOs with lower GC contents, the off-target effects seemed unavoidable. This prompted us to quantify the binding affinities between the t_{splice} MO and its canonical target and one of its putative off-target sites in *dtymk* causing alternative splicing (Figure 7D; Table S6). Primarily this was a comparison of the hybridization strength generated by 25 (target) and 8 (off-target) consecutive Watson-Crick base pairings. At both 23°C and 35°C biolayer interferometry yielded a ~100-fold lower affinity for the off-target than the canonical target RNA sequence: the MO concentrations at which half of the canonical sites were hybridized (equilibrium dissociation constant K_d) were ~14.3 and ~50.1 nM versus ~0.18 and 0.46 nM, respectively. The off-target affinities were comparable with that of the sequence-specific transcription factor Brachyury for its canonical DNA binding motif (Gentsch et al., 2013). Furthermore, wobble base pairing, here in the form of G:T, could contribute to the stability of hybridization as a G-to-C transversion increased the K_d by ~30%. No affinity was detected between a scrambled version of the off-target RNA sequence and the t_{splice} MO. These off-target K_d values were about 10-fold below the minimal concentration required to produce a *t/t2* KO-like phenotype, which was ~300 nM based on 0.75 ng of the t_{splice} MO being injected as part of the *t/t2* MO mix into a *X. tropicalis* zygote with a diameter of 0.8 mm. Therefore, we also conclude from a kinetic point view that these off-target effects are probably inevitable under physiological conditions.

DISCUSSION

Using TALEN technology, we generated $t^{-/+}t2^{+/-}$ *X. tropicalis* frogs, a double heterozygous line from which *Brachyury* null mutants were derived. This genetic KO was compared on a transcriptome-wide level with a previously validated MO-mediated KD (Gentsch et al., 2013). The main objective of this study was to ask whether morphants with KO-like characteristics are suitable for molecular profiling, since genetic null mutants may not be readily available in sufficient quantities or cannot be generated for other reasons (see Introduction). A gross comparison of the morphology and various mesoderm cell lineage markers showed no apparent differences between null mutants and morphants: In both cases—irrespective of the method of genetic interference—the loss of Brachyury proteins disrupted tissue-specific gene expression in the mesoderm and caused a severe truncation of the tail. This is consistent with previous observations in *Xenopus* (Gentsch et al., 2013) and other vertebrate embryos (Chesley, 1935; Grüneberg, 1958; Halpern et al., 1993; Martin and Kimelman, 2008; Schulte-Merker et al., 1994; Yamaguchi et al., 1999; Yanagisawa et al., 1981). However, a deeper analysis of the poly(A) transcriptome revealed that, while KO and KD equally affected the same neuromesodermal genes, including functional Brachyury targets, morphants differed significantly from their genetic counterparts. This was due to at least two different kinds of side effect observed with both *t/t2* MOs and the standard cMO.

Innate Immune Response against MOs

No immune stimulatory activity, such as interferon production or B cell activation has yet been attributed to MOs, in part because of their neutral chemistry (Moulton, 2016; Summerton, 2007). However, we show here that MOs can trigger such a response in *Xenopus* embryos, as judged by the perturbation of ~650 genes associated with the innate immune system. A spatiotemporal analysis of a few immune response related genes suggests that all embryonic cells are sensitive to MOs in this respect. This cell-intrinsic reaction was first detected during neurulation and intensified during axial elongation. The list of induced genes includes sensors, transducers, and effectors of innate immunity (Liu and Cao, 2016; Reis E Sousa, 2017; Takeuchi and Akira, 2010), suggesting that MOs are recognized by PRRs, such as specific TLRs, which induce NF-κB transcription factors and MAP kinases through the TLR signal mediator MyD88. These in turn activate the complement component system and release pro-inflammatory cytokines and protective molecules such as Δ99tp53. We suggest that this MO-induced immune response might generate non-specific abnormalities, especially in later development and in tissues and organ systems that co-opt signaling and gene regulatory networks from the immune system, such as the migrating neural crest and the cardiovascular and nervous systems. We note that the immune response appeared stronger for *t/t2* MOs than cMO, suggesting that, for transcriptional analyses and other molecular profiling techniques, the use of a cMO for comparisons is not enough to prevent erroneous results. We found that this discrepancy in immune reaction intensity was probably based on the higher GC content of *t/t2* MOs, in particular that of *t* MO_{transl} (65%) compared with cMO (32%). This is reminiscent of the immune response against unmethylated CpG DNA, a pathogen-associated physical pattern of bacteria and viruses being recognized by TLR9 in B cells and plasmacytoid dentritic cells (Hemmi et al., 2000;

(E) Multi-probe WMISH for various mesoderm cell lineage and derivative markers and single WMISH for the immune response related genes *tp53* and *c3ar1* in various late tailbud embryos developed at 25°C–26°C. Injection of the *t/t2* MO mix at 3 ng per embryo (3-fold above an inefficient dose of 1 ng) caused KO-like loss of *tbx6* and *cav1* expression domains, as indicated by white arrowheads, while keeping immune response at minimum.

(F) Dosage-dependent effects on mis-splicing and transcript fold changes (log_2 scale) in cMO- and *t/t2* MO-injected versus uninjected embryos quantified by qRT-PCR at early gastrula (st. 10^+) and mid-tailbud (st. 26) stage (n = 3, mean ± SD).

One-way ANOVA test: ***p < 0.001. Scale bars, 0.5 mm (B and E).

Krieg et al., 1995). Our study suggests that *Xenopus* post-gastrula embryos have similar immunological capacities *nota bene* in the absence of any specialized immune cells.

MO Off-Target Splicing Defects

MO off-target effects have previously been estimated to be rare, because 13–15 contiguous base matches (the minimum inactivating length, or MIL) have been thought to be necessary between the MO and RNA in order that splicing or translation be affected (Summerton, 1999, 2003, 2007). However, we detected hundreds of alternative or cryptic splice events that were probably due to the MO-mediated interference with the spliceosome-recognizing core RNA motifs of the splice or branchpoint sites or with auxiliary splicing factors binding additional pre-mRNA *cis*-acting sequences such as splicing enhancers or silencers (Scotti and Swanson, 2016). The branchpoint initiates splicing by forming an intron lariat with the 5′ end of the intron and determines the location of the acceptor splice site, which is normally located <50 bases 3′ of the branchpoint (Mercer et al., 2015). Mutations at branchpoint or splice sites can cause mis-splicing and are associated with various human genetic diseases like β-thalassemia or several muscular dystrophies (Scotti and Swanson, 2016; Singh and Cooper, 2012). There are at least three reasons that a higher rate of aberrant splicing in morphants might occur.

First, because the standard cMO as well as our target-specific splice-blocking MOs happened to match the most frequently encountered canonical donor splice motif in *Xenopus* and other vertebrates, they were more likely to show splice-related off-target effects. This would also include MOs targeting canonical acceptor splice sites which share similar sequence conservation (Scotti and Swanson, 2016). However, we show that splicing defects occur with all MOs tested, including translation-blocking MOs. Second, effective hybridization can tolerate a few interspersed mismatches between the MO and RNA (Kamachi et al., 2008). We confirm that non-canonical base pairing between guanine and thymine can stabilize hybridization (Moulton, 2017; Varani and McClain, 2000). These imperfect interactions including mismatches have also been observed between several small non-coding RNA species and mRNA transcripts (Martin et al., 2014; Mercer et al., 2015). Third, experimental *in vivo* conditions such as lower temperatures and higher MO molarities could reduce stringency (Coffman et al., 2004; Eisen and Smith, 2008). The MIL was determined in a cell-free translation system with 300 nM MO at 37°C (Summerton, 2003), while zebrafish and *X. tropicalis* embryos are routinely raised with higher MO concentrations at 22°C to 30°C. However, our *in vivo* experiments show that an increase of more than 6°C does not effectively alleviate mis-splicing, which is in accordance with the *in vitro* hybridization kinetics measured at 23°C and 35°C. MOs avidly hybridize off-target RNA oligonucleotides with a complementary sequence of only 8 consecutive bases at a concentration that is substantially lower than the absolute minimum of ~300 nM per MO required to produce a *t/t2* KO-like phenotype. As a result, widespread off-target MO hybridization across the entire transcriptome explains why despite their high affinity for the target sequence (~200 pM) MOs have to be injected in >1,000-fold excess to achieve an efficient KD.

MO-induced aberrations in splicing might affect transcript stability by removing or adding regulatory elements, perhaps by inducing usage of an alternative final exon or by introducing premature translation stop codons, which could subject the mis-spliced transcript to nonsense-mediated decay (Lykke-Andersen and Jensen, 2015). The number of MO off-target effects will be higher than we find here, because so far we have only investigated splicing, and left MO-induced defects in transcript stability and translation unexplored. This might be further analyzed by profiling poly(A) tails and ribosome footprints (Subtelny et al., 2014). It was beyond the scope of this study to predict off-target effects on a global scale. Several parameters seem likely to be important for modeling this: alignment stringency with regard to MIL including the allowance of wobble base pairing or mismatches, MO positioning on the transcript, MO molarity and temperature of hybridization.

Mitigation of MO Side Effects

We found that by reducing the MO dose its side effects—that is the immune response and off-target splicing defects—could be somewhat alleviated while retaining the *Brachyury* phenotype. With regard to the immune reaction, we estimate that most of it could be avoided by designing MOs with a GC content of ≤40% and performing MO dosage optimization. Unfortunately, these measurements including the elevation of the incubation temperature were not effective in neutralizing mis-splicing. This is in line with the high affinity of MO off-target hybridization measured *in vitro*. Nevertheless, maximal temperatures are desirable, as less MO was required for an efficient *Brachyury* KD at 28.5°C compared with 22°C. This is possibly because more MO oligomers were released from low-stringency hybridization and became available for primary gene interference. Our kinetic and transcriptional study suggests that MO side effects are likely even in the absence of any obvious macro- or microscopic deviations from a null phenotype, which is still considered the golden standard for MO use (Stainier et al., 2017). These side effects may be lower in pre-midblastula transition embryos as transcriptional/post-transcriptional regulation are not as pervasive as in later development.

Given the persistence of MO side effects, one may contemplate whether they could be discriminated *in silico* from the molecular changes causing the loss-of-function phenotype. In our case these MO side effects appear orthogonal to the BP under investigation, which is the perturbation of the neuromesodermal cell trajectory. However, we would refrain from using an MO-mediated KD to explore BPs related to the MO side effects such as alternative splicing or immune-related signaling pathways. In any case, the standard cMO is inappropriate for differential expression analysis as it creates its own sequence-specific off-target effects and may not be as immunogenic as the target MO due to its exceptionally low GC content. We recommend the use of uninjected embryos instead, and keeping the GC content and dosage of the target MO as low as possible to reduce MO side effects and any potential developmental delays. We conclude that, despite a superficial agreement with the KO phenotype, the deep molecular profiling of morphants requires careful attention with regard to MO-mediated immunogenic and off-target effects. These anomalies might also have far-reaching deleterious consequences when considering applying MOs therapeutically to correct human genetic splicing

defects (Cirak et al., 2011; Mendell et al., 2013; Scotti and Swanson, 2016; Summerton, 2017).

STAR★METHODS

Detailed methods are provided in the online version of this paper and include the following:

- KEY RESOURCES TABLE
- CONTACT FOR REAGENT AND RESOURCE SHARING
- EXPERIMENTAL MODEL AND SUBJECT DETAILS
  - Xenopus
- METHOD DETAILS
  - TALEN Design and Production
  - Morpholino and TALEN Injections
  - Genotyping
  - Cloning of Wild-Type and Mutant *t* and *t2*
  - Western Blotting
  - Total RNA Isolation
  - RNA-Seq Library Preparation and Sequencing
  - RNA-Seq Differential Expression Analysis
  - Perturbation Networks of Biological Processes
  - Visualization of Chromatin and RNA Profiles
  - Analysis of Differential Splicing
  - Generation of Digoxigenin-Labeled RNA Probes
  - Whole Mount *In Situ* Hybridization
  - Visualizing Apoptosis by TUNEL Staining
  - Quantification of Transcription and Splicing
  - Measurement of Morpholino:RNA Hybridization Affinity and Kinetics
- QUANTIFICATION AND STATISTICAL ANALYSIS
- DATA AND SOFTWARE AVAILABILITY

SUPPLEMENTAL INFORMATION

Supplemental Information includes five figures and six tables and can be found with this article online at https://doi.org/10.1016/j.devcel.2018.01.022.

ACKNOWLEDGMENTS

We thank Lyle Zimmerman and Tosikazu Amano for helping with the *X. tropicalis* frogs, and the Aquatics and Advanced Sequencing Facility for frog maintenance and sequencing; Mareike Thompson, Andreia Bernardo, and Victor Tybulewicz for critical reading of the manuscript; and the Smith lab for discussions and advice. G.E.G. and J.C.S. are supported by the Francis Crick Institute, which receives its core funding from Cancer Research UK (FC001-157), the UK Medical Research Council (FC001-157), and the Wellcome Trust (FC001-157). R.S.M. is supported by DevCom, a European Initial Training Network for Developmental and Computational Biology (Project Reference 607142).

AUTHOR CONTRIBUTIONS

G.E.G. and T.S. conceived and designed the experiments. T.S. generated the *t/t2* KO lines. R.S.M. genotyped and functionally verified the null mutations. G.E.G. investigated the splicing defects and the immune response. S.R.M. carried out biolayer interferometry. G.E.G. and N.D.L.O. performed the computational analysis of sequencing data. G.E.G. and J.C.S. supervised the project. G.E.G. wrote the paper. All authors were involved in revising the paper.

DECLARATION OF INTERESTS

The authors declare no competing interests.

Received: July 19, 2017
Revised: November 21, 2017
Accepted: January 26, 2018
Published: February 22, 2018

REFERENCES

Abu-Daya, A., Sater, A.K., Wells, D.E., Mohun, T.J., and Zimmerman, L.B. (2009). Absence of heartbeat in the *Xenopus tropicalis* mutation muzak is caused by a nonsense mutation in cardiac myosin myh6. Dev. Biol. *336*, 20–29.

Anders, S., Pyl, P.T., and Huber, W. (2015). HTSeq–a Python framework to work with high-throughput sequencing data. Bioinformatics *31*, 166–169.

Blum, M., De Robertis, E.M., Wallingford, J.B., and Niehrs, C. (2015). Morpholinos: antisense and sensibility. Dev. Cell *35*, 145–149.

Bogdanović, O., Smits, A.H., de la Calle Mustienes, E., Tena, J.J., Ford, E., Williams, R., Senanayake, U., Schultz, M.D., Hontelez, S., van Kruijsbergen, I., et al. (2016). Active DNA demethylation at enhancers during the vertebrate phylotypic period. Nat. Genet. *48*, 417–426.

Bourdon, J.-C., Fernandes, K., Murray-Zmijewski, F., Liu, G., Diot, A., Xirodimas, D.P., Saville, M.K., and Lane, D.P. (2005). p53 isoforms can regulate p53 transcriptional activity. Genes Dev. *19*, 2122–2137.

Campbell, E.P., Quigley, I.K., and Kintner, C. (2016). Foxn4 promotes gene expression required for the formation of multiple motile cilia. Development *143*, 4654–4664.

Carlson, D.F., Tan, W., Lillico, S.G., Stverakova, D., Proudfoot, C., Christian, M., Voytas, D.F., Long, C.R., Whitelaw, C.B.A., and Fahrenkrug, S.C. (2012). Efficient TALEN-mediated gene knockout in livestock. Proc. Natl. Acad. Sci. USA *109*, 17382–17387.

Cermak, T., Doyle, E.L., Christian, M., Wang, L., Zhang, Y., Schmidt, C., Baller, J.A., Somia, N.V., Bogdanove, A.J., and Voytas, D.F. (2011). Efficient design and assembly of custom TALEN and other TAL effector-based constructs for DNA targeting. Nucleic Acids Res. *39*, e82.

Chen, J., Ng, S.M., Chang, C., Zhang, Z., Bourdon, J.-C., Lane, D.P., and Peng, J. (2009). p53 isoform Δ113p53 is a p53 target gene that antagonizes p53 apoptotic activity via BclxL activation in zebrafish. Genes Dev. *23*, 278–290.

Chesley, P. (1935). Development of the short-tailed mutant in the house mouse. J. Exp. Zool. *70*, 429–459.

Chung, M.-I., Kwon, T., Tu, F., Brooks, E.R., Gupta, R., Meyer, M., Baker, J.C., Marcotte, E.M., and Wallingford, J.B. (2014). Coordinated genomic control of ciliogenesis and cell movement by RFX2. Elife *3*, e01439.

Cirak, S., Arechavala-Gomeza, V., Guglieri, M., Feng, L., Torelli, S., Anthony, K., Abbs, S., Garralda, M.E., Bourke, J., Wells, D.J., et al. (2011). Exon skipping and dystrophin restoration in patients with Duchenne muscular dystrophy after systemic phosphorodiamidate morpholino oligomer treatment: an open-label, phase 2, dose-escalation study. Lancet *378*, 595–605.

Coffman, J.A., Dickey-Sims, C., Haug, J.S., McCarthy, J.J., and Robertson, A.J. (2004). Evaluation of developmental phenotypes produced by morpholino antisense targeting of a sea urchin Runx gene. BMC Biol. *2*, 6.

Collart, C., Christov, C.P., Smith, J.C., and Krude, T. (2011). The midblastula transition defines the onset of Y RNA-dependent DNA replication in *Xenopus laevis*. Mol. Cell Biol. *31*, 3857–3870.

Collart, C., Owens, N.D.L., Bhaw-Rosun, L., Cooper, B., De Domenico, E., Patrushev, I., Sesay, A.K., Smith, J.N., Smith, J.C., and Gilchrist, M.J. (2014). High-resolution analysis of gene activity during the *Xenopus* mid-blastula transition. Development *141*, 1927–1939.

Conesa, A., Götz, S., García-Gómez, J.M., Terol, J., Talón, M., and Robles, M. (2005). Blast2GO: a universal tool for annotation, visualization and analysis in functional genomics research. Bioinformatics *21*, 3674–3676.

Cordenonsi, M., Montagner, M., Adorno, M., Zacchigna, L., Martello, G., Mamidi, A., Soligo, S., Dupont, S., and Piccolo, S. (2007). Integration of TGF-beta and Ras/MAPK signaling through p53 phosphorylation. Science *315*, 840–843.

Crooks, G.E., Hon, G., Chandonia, J.-M., and Brenner, S.E. (2004). WebLogo: a sequence logo generator. Genome Res. *14*, 1188–1190.

Csárdi, G., and Nepusz, T. (2006). The igraph software package for complex network research. InterJournal Complex Systems, 1695. http://igraph.org.

Dichmann, D.S., Walentek, P., and Harland, R.M. (2015). The alternative splicing regulator Tra2b is required for somitogenesis and regulates splicing of an inhibitory Wnt11b isoform. Cell Rep. *10*, 527–536.

Dobin, A., Davis, C.A., Schlesinger, F., Drenkow, J., Zaleski, C., Jha, S., Batut, P., Chaisson, M., and Gingeras, T.R. (2013). STAR: ultrafast universal RNA-seq aligner. Bioinformatics *29*, 15–21.

Doyle, E.L., Booher, N.J., Standage, D.S., Voytas, D.F., Brendel, V.P., Vandyk, J.K., and Bogdanove, A.J. (2012). TAL Effector-Nucleotide Targeter (TALE-NT) 2.0: tools for TAL effector design and target prediction. Nucleic Acids Res. *40*, W117–W122.

Eisen, J.S., and Smith, J.C. (2008). Controlling morpholino experiments: don't stop making antisense. Development *135*, 1735–1743.

Falcon, S., and Gentleman, R. (2007). Using GOstats to test gene lists for GO term association. Bioinformatics *23*, 257–258.

Gentsch, G.E., Owens, N.D.L., Martin, S.R., Piccinelli, P., Faial, T., Trotter, M.W.B., Gilchrist, M.J., and Smith, J.C. (2013). In vivo T-box transcription factor profiling reveals joint regulation of embryonic neuromesodermal bipotency. Cell Rep. *4*, 1185–1196.

Gentsch, G.E., Patrushev, I., and Smith, J.C. (2015). Genome-wide snapshot of chromatin regulators and states in *Xenopus* embryos by ChIP-Seq. J. Vis. Exp. *96*, e52535.

Grüneberg, H. (1958). Genetical studies on the skeleton of the mouse. XXIII. The development of brachyury and anury. J. Embryol. Exp. Morphol. *6*, 424–443.

Hahsler, M., Hornik, K., and Buchta, C. (2008). Getting things in order: an introduction to the r package seriation. J. Stat. Softw. *25*, https://doi.org/10.18637/jss.v025.i03.

Halpern, M., Ho, R., Walker, C., and Kimmel, C. (1993). Induction of muscle pioneers and floor plate is distinguished by the zebrafish no tail mutation. Cell *75*, 99–111.

Hayata, T., Eisaki, A., Kuroda, H., and Asashima, M. (1999). Expression of Brachyury-like T-box transcription factor, Xbra3 in *Xenopus* embryo. Dev. Genes Evol. *209*, 560–563.

Heinz, S., Benner, C., Spann, N., Bertolino, E., Lin, Y.C., Laslo, P., Cheng, J.X., Murre, C., Singh, H., and Glass, C.K. (2010). Simple combinations of lineage-determining transcription factors prime *cis*-regulatory elements required for macrophage and B cell identities. Mol. Cell *38*, 576–589.

Hemmi, H., Takeuchi, O., Kawai, T., Kaisho, T., Sato, S., Sanjo, H., Matsumoto, M., Hoshino, K., Wagner, H., Takeda, K., et al. (2000). A Toll-like receptor recognizes bacterial DNA. Nature *408*, 740–745.

Hensey, C., and Gautier, J. (1998). Programmed cell death during *Xenopus* development: a spatio-temporal analysis. Dev. Biol. *203*, 36–48.

Kahles, A., Ong, C.S., Zhong, Y., and Ratsch, G. (2016). SplAdder: identification, quantification and testing of alternative splicing events from RNA-seq data. Bioinformatics *32*, 1840–1847.

Kamachi, Y., Okuda, Y., and Kondoh, H. (2008). Quantitative assessment of the knockdown efficiency of morpholino antisense oligonucleotides in zebrafish embryos using a luciferase assay. Genesis *46*, 1–7.

Kang, S.H., Cho, M.J., and Kole, R. (1998). Up-regulation of luciferase gene expression with antisense oligonucleotides: implications and applications in functional assay development. Biochemistry *37*, 6235–6239.

Khokha, M., Chung, C., Bustamante, E., Gaw, L., Trott, K., Yeh, J., Lim, N., Lin, J., Taverner, N., Amaya, E., et al. (2002). Techniques and probes for the study of *Xenopus tropicalis* development. Dev. Dyn. *225*, 499–510.

Khokha, M., Yeh, J., Grammer, T., and Harland, R. (2005). Depletion of three BMP antagonists from Spemann's organizer leads to a catastrophic loss of dorsal structures. Dev. Cell *8*, 401–411.

Khoury, M.P., and Bourdon, J.-C. (2010). The isoforms of the p53 protein. Cold Spring Harb. Perspect. Biol. *2*, a000927.

Kim, D., Pertea, G., Trapnell, C., Pimentel, H., Kelley, R., and Salzberg, S.L. (2013). TopHat2: accurate alignment of transcriptomes in the presence of insertions, deletions and gene fusions. Genome Biol. *14*, R36.

Kloosterman, W.P., Lagendijk, A.K., Ketting, R.F., Moulton, J.D., and Plasterk, R.H.A. (2007). Targeted inhibition of miRNA maturation with morpholinos reveals a role for miR-375 in pancreatic islet development. PLoS Biol. *5*, e203.

Kok, F.O., Shin, M., Ni, C.-W., Gupta, A., Grosse, A.S., van Impel, A., Kirchmaier, B.C., Peterson-Maduro, J., Kourkoulis, G., Male, I., et al. (2015). Reverse genetic screening reveals poor correlation between morpholino-induced and mutant phenotypes in zebrafish. Dev. Cell *32*, 97–108.

Krieg, A.M., Yi, A.K., Matson, S., Waldschmidt, T.J., Bishop, G.A., Teasdale, R., Koretzky, G.A., and Klinman, D.M. (1995). CpG motifs in bacterial DNA trigger direct B-cell activation. Nature *374*, 546–549.

Lane, D.P. (1992). Cancer. p53, guardian of the genome. Nature *358*, 15–16.

Langmead, B., and Salzberg, S.L. (2012). Fast gapped-read alignment with Bowtie 2. Nat. Methods *9*, 357–359.

Li, H., Handsaker, B., Wysoker, A., Fennell, T., Ruan, J., Homer, N., Marth, G., Abecasis, G., and Durbin, R.; 1000 Genome Project Data Processing Subgroup (2009). The Sequence Alignment/Map format and SAMtools. Bioinformatics *25*, 2078–2079.

Li, Y.I., Knowles, D.A., Humphrey, J., Barbeira, A.N., Dickinson, S.P., Im, H.K., and Pritchard, J.K. (2018). Annotation-free quantification of RNA splicing using LeafCutter. Nat. Genet. *50*, 151–158.

Liu, J., and Cao, X. (2016). Cellular and molecular regulation of innate inflammatory responses. Cell Mol. Immunol. *13*, 711–721.

Liu, Y., Zhu, Z., Ho, I.H.T., Shi, Y., Xie, Y., Li, J., Zhang, Y., Chan, M.T.V., and Cheng, C.H.K. (2017). Germline-specific dgcr8 knockout in zebrafish using a BACK approach. Cell Mol. Life Sci. *74*, 2503–2511.

Livak, K.J., and Schmittgen, T.D. (2001). Analysis of relative gene expression data using real-time quantitative PCR and the 2CT method. Methods *25*, 402–408.

Love, N.R., Chen, Y., Ishibashi, S., Kritsiligkou, P., Lea, R., Koh, Y., Gallop, J.L., Dorey, K., and Amaya, E. (2013). Amputation-induced reactive oxygen species are required for successful *Xenopus* tadpole tail regeneration. Nat. Cell Biol. *15*, 222–228.

Love, M.I., Huber, W., and Anders, S. (2014). Moderated estimation of fold change and dispersion for RNA-seq data with DESeq2. Genome Biol. *15*, 550.

Lykke-Andersen, S., and Jensen, T.H. (2015). Nonsense-mediated mRNA decay: an intricate machinery that shapes transcriptomes. Nat. Rev. Mol. Cell Biol. *16*, 665–677.

Marlétaz, F., Maeso, I., Faas, L., Isaacs, H.V., and Holland, P.W.H. (2015). Cdx ParaHox genes acquired distinct developmental roles after gene duplication in vertebrate evolution. BMC Biol. *13*, 56.

Martin, B.L., and Kimelman, D. (2008). Regulation of canonical wnt signaling by brachyury is essential for posterior mesoderm formation. Dev. Cell *15*, 121–133.

Martin, H.C., Wani, S., Steptoe, A.L., Krishnan, K., Nones, K., Nourbakhsh, E., Vlassov, A., Grimmond, S.M., and Cloonan, N. (2014). Imperfect centered miRNA binding sites are common and can mediate repression of target mRNAs. Genome Biol. *15*, R51.

Mendell, J.R., Rodino-Klapac, L.R., Sahenk, Z., Roush, K., Bird, L., Lowes, L.P., Alfano, L., Gomez, A.M., Lewis, S., Kota, J., et al. (2013). Eteplirsen for the treatment of Duchenne muscular dystrophy. Ann. Neurol. *74*, 637–647.

Mercer, T.R., Clark, M.B., Andersen, S.B., Brunck, M.E., Haerty, W., Crawford, J., Taft, R.J., Nielsen, L.K., Dinger, M.E., and Mattick, J.S. (2015). Genome-wide discovery of human splicing branchpoints. Genome Res. *25*, 290–303.

Mohun, T., Brennan, S., Dathan, N., Fairman, S., and Gurdon, J. (1984). Cell type-specific activation of actin genes in the early amphibian embryo. Nature *311*, 716–721.

Monsoro-Burq, A.H. (2007). A rapid protocol for whole-mount in situ hybridization on xenopus embryos. CSH Protoc. *2007*, pdb.prot4809.

Morgan, M., Falcon, S., and Gentleman, R. (2017). GSEABase: gene set enrichment data structures and methods.

Moulton, J.D. (2016). Guide for morpholino users: toward therapeutics. J. Drug Discov. Dev. Deliv. *3*, 1023.

Moulton, J.D. (2017). Making a morpholino experiment work: controls, favoring specificity, improving efficacy, storage, and dose. Methods Mol. Biol. *1565*, 17–29.

Mülleder, M., Calvani, E., Alam, M.T., Wang, R.K., Eckerstorfer, F., Zelezniak, A., and Ralser, M. (2016). Functional metabolomics describes the yeast biosynthetic regulome. Cell *167*, 553–565.

Nakamura, Y., de Paiva Alves, E., Veenstra, G.J.C., and Hoppler, S. (2016). Tissue- and stage-specific Wnt target gene expression is controlled subsequent to β-catenin recruitment to cis-regulatory modules. Development *143*, 1914–1925.

Nieuwkoop, P., and Faber, J. (1994). Normal Table of *Xenopus laevis* (Daudin): A Systematical and Chronological Survey of the Development from the Fertilized Egg till the End of Metamorphosis (Garland).

Noiret, M., Mottier, S., Angrand, G., Gautier-Courteille, C., Lerivray, H., Viet, J., Paillard, L., Mereau, A., Hardy, S., and Audic, Y. (2016). Ptbp1 and Exosc9 knockdowns trigger skin stability defects through different pathways. Dev. Biol. *409*, 489–501.

Owens, N.D.L., Blitz, I.L., Lane, M.A., Patrushev, I., Overton, J.D., Gilchrist, M.J., Cho, K.W.Y., and Khokha, M.K. (2016). Measuring absolute RNA copy numbers at high temporal resolution reveals transcriptome kinetics in development. Cell Rep. *14*, 632–647.

Reis E Sousa, C. (2017). Sensing infection and tissue damage. EMBO Mol. Med. *9*, 285–288.

Reversade, B., Kuroda, H., Lee, H., Mays, A., and De Robertis, E.M. (2005). Depletion of Bmp2, Bmp4, Bmp7 and Spemann organizer signals induces massive brain formation in *Xenopus* embryos. Development *132*, 3381–3392.

Ritchie, M.E., Phipson, B., Wu, D., Hu, Y., Law, C.W., Shi, W., and Smyth, G.K. (2015). Limma powers differential expression analyses for RNA-sequencing and microarray studies. Nucleic Acids Res. *43*, e47.

Robinson, M.D., and Oshlack, A. (2010). A scaling normalization method for differential expression analysis of RNA-seq data. Genome Biol. *11*, R25.

Robinson, M.D., McCarthy, D.J., and Smyth, G.K. (2010). edgeR: a Bioconductor package for differential expression analysis of digital gene expression data. Bioinformatics *26*, 139–140.

Robinson, J.T., Thorvaldsdóttir, H., Winckler, W., Guttman, M., Lander, E.S., Getz, G., and Mesirov, J.P. (2011). Integrative genomics viewer. Nat. Biotechnol. *29*, 24–26.

Robu, M.E., Larson, J.D., Nasevicius, A., Beiraghi, S., Brenner, C., Farber, S.A., and Ekker, S.C. (2007). p53 activation by knockdown technologies. PLoS Genet. *3*, e78.

Rossi, A., Kontarakis, Z., Gerri, C., Nolte, H., Hölper, S., Krüger, M., and Stainier, D.Y.R. (2015). Genetic compensation induced by deleterious mutations but not gene knockdowns. Nature *524*, 230–233.

Schulte-Merker, S., van Eeden, F., Halpern, M., Kimmel, C., and Nusslein-Volhard, C. (1994). No tail (ntl) is the zebrafish homologue of the mouse T (Brachyury) gene. Development *120*, 1009–1015.

Scotti, M.M., and Swanson, M.S. (2016). RNA mis-splicing in disease. Nat. Rev. Genet. *17*, 19–32.

Sekhon, J.S. (2011). Multivariate and propensity score matching software with automated balance optimization: the Matching package for R. J. Stat. Softw. *42*, https://doi.org/10.18637/jss.v042.i07.

Singh, R.K., and Cooper, T.A. (2012). Pre-mRNA splicing in disease and therapeutics. Trends Mol. Med. *18*, 472–482.

Sive, H., Grainger, R., and Harland, R. (2000). Early Development of *Xenopus laevis*: A Laboratory Manual (Cold Spring Harbor Laboratory Press).

Stainier, D.Y.R., Raz, E., Lawson, N.D., Ekker, S.C., Burdine, R.D., Eisen, J.S., Ingham, P.W., Schulte-Merker, S., Yelon, D., Weinstein, B.M., et al. (2017). Guidelines for morpholino use in zebrafish. PLoS Genet. *13*, e1007000.

Subtelny, A.O., Eichhorn, S.W., Chen, G.R., Sive, H., and Bartel, D.P. (2014). Poly(A)-tail profiling reveals an embryonic switch in translational control. Nature *508*, 66–71.

Summerton, J. (1999). Morpholino antisense oligomers: the case for an RNase H-independent structural type. Biochim. Biophys. Acta *1489*, 141–158.

Summerton, J. (2003). Morpholinos and PNAs compared. Lett. Pept. Sci. *10*, 215–236.

Summerton, J.E. (2007). Morpholino, siRNA, and S-DNA compared: impact of structure and mechanism of action on off-target effects and sequence specificity. Curr. Top. Med. Chem. *7*, 651–660.

Summerton, J.E. (2017). Invention and early history of morpholinos: from pipe dream to practical products. Methods Mol. Biol. *1565*, 1–15.

Takeuchi, O., and Akira, S. (2010). Pattern recognition receptors and inflammation. Cell *140*, 805–820.

Trindade, M., Messenger, N., Papin, C., Grimmer, D., Fairclough, L., Tada, M., and Smith, J.C. (2003). Regulation of apoptosis in the*Xenopus* embryo by Bix3. Development *130*, 4611–4622.

Uchiyama, H., Kobayashi, T., Yamashita, A., Ohno, S., and Yabe, S. (2001). Cloning and characterization of the T-box gene Tbx6 in *Xenopus laevis*. Dev. Growth Differ. *43*, 657–669.

Varani, G., and McClain, W.H. (2000). The G x U wobble base pair. A fundamental building block of RNA structure crucial to RNA function in diverse biological systems. EMBO Rep. *1*, 18–23.

Wang, L., Wang, S., and Li, W. (2012). RSeQC: quality control of RNA-seq experiments. Bioinformatics *28*, 2184–2185.

Yamaguchi, T.P., Takada, S., Yoshikawa, Y., Wu, N., and McMahon, A.P. (1999). T (Brachyury) is a direct target of Wnt3a during paraxial mesoderm specification. Genes Dev. *13*, 3185–3190.

Yanagisawa, K.O., Fujimoto, H., and Urushihara, H. (1981). Effects of the brachyury (T) mutation on morphogenetic movement in the mouse embryo. Dev. Biol. *87*, 242–248.

Yasuoka, Y., Suzuki, Y., Takahashi, S., Someya, H., Sudou, N., Haramoto, Y., Cho, K.W., Asashima, M., Sugano, S., and Taira, M. (2014). Occupancy of tissue-specific cis-regulatory modules by Otx2 and TLE/Groucho for embryonic head specification. Nat. Commun. *5*, 4322.

STAR★METHODS

KEY RESOURCES TABLE

REAGENT or RESOURCE	SOURCE	IDENTIFIER
Antibodies		
Mouse monoclonal anti-HA	Sigma	Cat#H9658; RRID: AB_260092
Mouse monoclonal anti-c-Myc	Sigma	Cat#M5546; RRID: AB_260581
Mouse monoclonal anti-α-tubulin	Sigma	Cat#T5168; RRID: AB_477579
Anti-mouse IgG (H+L) horse radish peroxidase conjugate	Thermo Fisher Scientific	Cat#31430; RRID: AB_228307
Fab fragments from polyclonal anti-digoxigenin conjugated to alkaline phosphatase	Roche	Cat#11093274910; RRID: AB_514497
Chemicals, Peptides, and Recombinant Proteins		
PhosphoSafe extraction buffer	Merck	Cat#71296
complete EDTA-free protease inhibitors	Roche	Cat#11873580001
TRIzol	Thermo Fisher Scientific	Cat#15596018
digoxigenin-11-UTP	Roche	Cat#11277065910
RiboLock RNase inhibitor	Thermo Fisher Scientific	Cat#EO0381
4-Nitro-blue tetrazolium chloride (NBT)	Roche	Cat#11383213001
5-Bromo-4-chloro-3’-indolyphosphate (BCIP)	Roche	Cat#11383221001
digoxigenin-11-dUTP	Roche	Cat#11558706910
Critical Commercial Assays		
mMessage mMachine T3 Transcription kit	Thermo Fisher Scientific	Cat#AM1348
mMessage mMachine SP6 Transcription kit	Thermo Fisher Scientific	Cat#AM1340
TruSeq RNA Library Prep Kit v2	Illumina	Cat#RS-122-2001
KAPA HiFi HotStart ReadyMix	Kapa Biosystems	Cat#KK2602
TOPO TA cloning kit	Thermo Fisher Scientific	Cat#K4500
Zero-Blunt TOPO cloning kit	Thermo Fisher Scientific	Cat#K2800
Directional pENTR/TOPO cloning kit	Thermo Fisher Scientific	Cat#K2400
Turbo DNase	Thermo Fisher Scientific	Cat#AM2238
proteinase K	Thermo Fisher Scientific	Cat#AM2548
SP6 RNA polymerase	Roche	Cat#11487671001
T7 RNA polymerase	Roche	Cat#10881767001
Terminal deoxynucleotidyl transferase	Thermo Fisher Scientific	Cat#EP0161
RNase H minus and point-mutant M-MLV reverse transcriptase	Promega	Cat#M3681
SYBR Green I master mix	Roche	Cat#04707516001
Deposited Data		
Raw sequencing data (FASTQ)	This study	GEO: GSE96655
Experimental Models: Organisms/Strains		
Xenopus tropicalis $t^{e1.2D/+}$	This study	EXRC: https://xenopusresource.org
Xenopus tropicalis $t^{e1.2D/+}t2^{e3.7D/+}$	This study	EXRC: https://xenopusresource.org
Oligonucleotides		
Sulforhodamine-tagged morpholino, *t* MO_{splice}: TGGAGAGACCCTGATCTTACCTTCC	GeneTools	Gentsch et al., 2013
Sulforhodamine-tagged morpholino, *t* MO_{transl}: GGCTTCCAAGCGCACACACTGGG	GeneTools	Gentsch et al., 2013
Sulforhodamine-tagged morpholino, *t2* MO_{splice}: GAAAGGTCCATATTCTCTTACCTTC	GeneTools	Gentsch et al., 2013

(*Continued on next page*)

Continued

REAGENT or RESOURCE	SOURCE	IDENTIFIER
Sulforhodamine-tagged morpholino, *t2* MO_{transl}: AGCTGTGCCTGTGCTCATTGTATTG	GeneTools	Gentsch et al., 2013
Untagged morpholino, standard control MO: CCTCTTACCTCAGTTACAATTTATA	GeneTools	N/A
Biotin-tagged t_{target} RNA: [Btn]AAAAAAAAGGAAGGUAAGAUCAGGGUCUCUCCA	IDT	This study
Biotin-tagged $dtymk_{off\text{-}target}$ RNA: [Btn]AAAAAAAAGGAGGUAAGAGAUCAUUUGUAGGAU	IDT	This study
Biotin-tagged $dtymk^{G>C}{}_{off\text{-}target}$ RNA: [Btn]AAAAAAAAG**C**AGGUAAGAGAUCAUUUGUAGGAU	IDT	This study
Biotin-tagged scrambled $dtymk_{off\text{-}target}$ RNA: [Btn]AAAAAAAAAGUUAUGGCGAGAGUGGAUAUUAGA	IDT	This study
Genotyping *t* (exon 1), forward and reverse primer: AATCAGAGGAAGAGCTGCTG, CATTGGTGAGCTCCTTGAAC	Sigma	This study
Genotyping *t2* (exon 3), forward and reverse primer: TCACATCATTAAAATAGTGGCCTGCT, TCCATGAAATGTGAATTGTGGGCT	Sigma	This study
Cloning *t* and $t^{e1.2D}$ for N-terminal tagging, forward and reverse primer: CACCATGAGTGTAAGTGCCACCGAGA, TTAGATTGACGGCGGTGCAAC	Sigma	This study
Cloning *t* and $t^{e1.2D}$ for C-terminal tagging, forward and reverse primer: CACCATGAGTGTAAGTGCCACCGAGA, GATTGACGGCGGTGCAACTG	Sigma	This study
Cloning *t2* for N-terminal tagging, forward and reverse primer: CACCATGAGTACAGGAACAGCTG, CTATAATGATGGAGGTGTCACAGA	Sigma	This study
Cloning *t2* for C-terminal tagging, forward and reverse primer: CACCCAGAAGAGGCATCAGCAATAC, TAATGATGGAGGTGTCACAGAAG	Sigma	This study
Cloning $t2^{e3.7D}$ for N-terminal tagging, forward and reverse primer: CACCATGAGCACAGGCACAGCTGAGA, CTATAATGATGGAGGTGTCACAGA	Sigma	This study
Cloning $t2^{e3.7D}$ for C-terminal tagging, forward and reverse primer: CACCATGAGCACAGGCACAGCTGAGA, TAATGATGGAGGTGTCACAGAAGC	Sigma	This study
Generating *c3ar1 in situ* probe template (1,020 bp), forward and reverse primer: GAGAGAGTGCCCGTTACAGC, ATGAGGCAGTTTGTGCCAAG	Sigma	This study
Generating *tp53 in situ* probe template (999 bp), forward and reverse primer: TGTGGAGTCTGTTGCCTGAC, CCAGCAGCTTCTTTCCTTTC	Sigma	This study
Generating *tp53inp1 in situ* probe template (1,002 bp), forward and reverse primer: TCGTCTACCTCACCCGTTTC, TGCAAGTCGCTCTGCTACTC	Sigma	This study
RT-qPCR for *abi1* (exon 1), forward and reverse primer: CGGGTGTGGACTTAGCTGTT, CGGGGATCTCCTCCTCTAGT	Sigma	This study

(*Continued on next page*)

Continued

REAGENT or RESOURCE	SOURCE	IDENTIFIER
RT-qPCR for *abi1* (^, joining exon 7 and 11 by alternative splicing), forward and reverse primer: TATTGGACAAG^TTGCGGACA, GGAGGTGGAGGAGGAGAATC	Sigma	This study
RT-qPCR for *bloc1s4* (exon 1 to 2), forward and reverse primer: CCAGTCTCCTGACCGAAGAG, AATCTCTGCATTTCCGCTGT	Sigma	This study
RT-qPCR for *bloc1s4* (^, joining exon 4 and 6 by alternative splicing), forward and reverse primer: TGCTTGAG^AAACAAACACCTG, CTGCTGCTGGGAAAGAAATC	Sigma	This study
RT-qPCR for *c3ar1* (exon 1 to 2), forward and reverse primer: TTGATGGTCAGGAGACAGAGG, CGTCCCATTCCTGATATTGC	Sigma	This study
RT-qPCR for *dtymk* (exon 1), forward and reverse primer: TGCGAGGTGCTTTAATTGTG, CTTGTAACCCCGCTCTTTCA	Sigma	This study
RT-qPCR for *dtymk* (^, joining exon 2 and 5 by alternative splicing), forward and reverse primer: CGCTGGGAACAAGT^CTCATT, TCGGTTTATCTTTGGCATCC	Sigma	This study
RT-qPCR for *odc1*, forward and reverse primer: GGGCAAAAGAGCTTAATGTGG, CATCGTGCATCTGAGACAGC	Sigma	Gentsch et al., 2013
RT-qPCR for *t* (exon 1 to 2), forward and reverse primer: CCTGTGGATGAGGTTCAAGG, CACGCTCACCTTTAGAACTGG	Sigma	Gentsch et al., 2013
RT-qPCR for *t* (exon 6), forward and reverse primer: TCTTTATGTTCGCCCAATCC, CGAGCGGTGGTTTCTTAGAG	Sigma	This study
RT-qPCR for *t* (^, joining exon 1 and 2 by cryptic splicing), forward and reverse primer: GAGCTGAAG^GCGAATGTTTC, TTGTCAGCTGCCACAAAATC	Sigma	This study
RT-qPCR for *t2*, forward and reverse primer: CACAAGCCATTCATTTCCAG, TCTTTGGCATCAAGGAAAGC	Sigma	Gentsch et al., 2013
RT-qPCR for *tbx6*, forward and reverse primer: ACCTCCTACGGGATGAGACC, AACAGCCCATCAAACCTCTG	Sigma	Gentsch et al., 2013
RT-qPCR for *tp53*, forward and reverse primer: GGATCGTCGCACAGAAGAAG, AAGTGGAGGGTCACTGGATG	Sigma	This study
RT-qPCR for *tp53inp1*, forward and reverse primer: CACAGGATATAAAGCGACCAAAG, GTGTAGCAAGGTGGCACAAG	Sigma	This study
Recombinant DNA		
t TALEN	Cellectis Bioresearch	N/A
t2 TALEN	This study	N/A
Golden Gate TALEN and TAL Effector Kit 2.0	Cermak et al., 2011	Addgene: goldengatev2
t2 cDNA clone	Source BioScience	IMAGE 5307982

(*Continued on next page*)

Continued

REAGENT or RESOURCE	SOURCE	IDENTIFIER
RCIscript-GoldyTALEN plasmid	Carlson et al., 2012	Addgene: 38142
N-terminal 3xHA pCS2+ destination vector	Nigel Messenger	Smith lab
C-terminal 3xHA pCS2+ destination vector	Nigel Messenger	Smith lab
myc-tagged *fam83g* pCS2+	Kevin Dingwell	Smith lab
X. laevis tp53 pCS105	Cordenonsi et al., 2007	N/A
X. laevis actc1 pSP21	Mohun et al., 1984	N/A
X. tropicalis cav1 pExpress1	Gentsch et al., 2013	IMAGE: 7024946
X. tropicalis c3ar1 pCRII-TOPO	This study	N/A
X. tropicalis hoxd8 pCR2.1-TOPO	Gentsch et al., 2013	N/A
X. tropicalis myh6 pCRII-TOPO	Abu-Daya et al., 2009	N/A
X. laevis tal1 pGEM-7Zf+	N/A	EXRC
X. laevis tbx6 pBluescript KS-	Uchiyama et al., 2001	N/A
X. tropicalis tp53 pCRII-TOPO	This study	N/A
X. tropicalis tp53inp1 pCRII-TOPO	This study	N/A
Software and Algorithms		
TAL Effector Nucleotide Targeter 2.0	Cermak et al., 2011; Doyle et al., 2012	https://tale-nt.cac.cornell.edu
Bowtie2 v2.1.0	Langmead and Salzberg, 2012	http://bowtie-bio.sourceforge.net/bowtie2
Tophat v2.0.10	Kim et al., 2013	https://ccb.jhu.edu/software/tophat
STAR v2.5.2a	Dobin et al., 2013	https://github.com/alexdobin/STAR
Samtools v1.3.1	Li et al., 2009	http://www.htslib.org
RSeQC v2.6.4 (Python)	Wang et al., 2012	http://rseqc.sourceforge.net
IGV genome browser v2.3.92	Robinson et al., 2011	http://software.broadinstitute.org/software/igv/
HOMER v4.8.3	Heinz et al., 2010	http://homer.ucsd.edu/homer/index.html
LeafCutter v1.0 (Python/R package)	Li et al., 2018	https://github.com/davidaknowles/leafcutter
splAdder v1.0 (Python)	Kahles et al., 2016	https://github.com/ratschlab/spladder
Julia: Bio.Seq module	BioJulia Project	https://github.com/BioJulia
HTSeq-count v0.5.4p3 (Python)	Anders et al., 2015	http://www-huber.embl.de/HTSeq/doc/count.html
DESeq2 v1.14.1 (Bioconductor package)	Love et al., 2014	https://bioconductor.org/packages/release/bioc/html/DESeq2.html
limma v3.30.13 (Bioconductor package)	Ritchie et al., 2015	https://bioconductor.org/packages/release/bioc/html/limma.html
edgeR v3.16.5 (Bioconductor package)	Robinson et al., 2010	https://bioconductor.org/packages/release/bioc/html/edgeR.html
GOstats v2.40.0 (Bioconductor package)	Falcon and Gentleman, 2007	https://bioconductor.org/packages/release/bioc/html/GOstats.html
GSEABase v1.36.0 (Bioconductor package)	Morgan et al., 2017	http://bioconductor.org/packages/release/bioc/html/GSEABase.html
igraph v1.0.1 (R package)	Csárdi and Nepusz, 2006	https://cran.r-project.org/web/packages/igraph/index.html
seriation v1.2-1 (R package)	Hahsler et al., 2008	https://cran.r-project.org/web/packages/seriation/index.html
Matching v4.9-2 (R package)	Sekhon, 2011	https://cran.r-project.org/web/packages/Matching/index.html
R v3.3.1	The R Foundation	https://www.r-project.org
Bioconductor v3.5	Bioconductor	http://www.bioconductor.org
Perl v5.18.2	The Perl Foundation	https://www.perl.org
Python v2.7.12	Python Software Foundation	http://www.python.org
Julia v0.5	The Julia Community	https://julialang.org

CONTACT FOR REAGENT AND RESOURCE SHARING

Further information and requests for resources and reagents should be directed to and will be fulfilled by the Lead Contact, James C. Smith (jim.smith@crick.ac.uk).

EXPERIMENTAL MODEL AND SUBJECT DETAILS

Xenopus

Standard procedures were used for ovulation, fertilization, and manipulation and incubation of embryos (Khokha et al., 2002; Sive et al., 2000). Embryos were staged according to Nieuwkoop and Faber (1994). All *Xenopus* work complied fully with the UK Animals (Scientific Procedures) Act 1986 as implemented by the Francis Crick Institute.

METHOD DETAILS

TALEN Design and Production

Plasmids encoding the *t* targeting TALEN pair were purchased from Cellectis Bioresearch (France). The repeat-variable diresidues (RVDs) of the left (NG-HD-NG-HD-NI-NN-HD-NN-HD-NG-NN-NG-NN-NN-NI-NG) and the right (HD-HD-HD-HD-HD-NG-NG-HD-NG-HD-NN-HD-NG-NN-HD-NG) TALEN target the genomic DNA sequence 5’-TTCTCAGCGCTGTGGAG-3’ (*X. tropicalis* genome assembly v9.0, Chr05:58,625,117-58,625,133) and 5’-TCCCCCTTCTCGCTGCC-3’ (Chr05:58,625,085-58,625,085-58,625,101), respectively. The *t2* targeting TALEN pair was designed using TAL Effector Nucleotide Targeter 2.0 software (Cermak et al., 2011; Doyle et al., 2012) and constructed using the Golden Gate TALEN and TAL Effector Kit 2.0 (Cermak et al., 2011) (Addgene goldengatev2). The RVDs of the left (HD-NN-NG-NG-NN-HD-NI-NG-NI-NI-NN-NG-NI-NG-NN-NI-NI-HD-HD-HD) and right (NG-NG-HD-NG-NN-NG-NN-NN-NG-HD-HD-HD-HD-HD-NI-NI-HD-NG-HD-NG) *t2* TALEN target genomic DNA sequence 5’-CGTTGCATAA GTATGAACCC-3’ (Chr05:58,584,630-58,584,649) and 5’-AGAGTTGGGGGACCACAGAA-3’ (Chr05:58,584,595-58,584,614), respectively. The RCIscript-GoldyTALEN plasmid (Carlson et al., 2012) (Addgene, Cat#38142) was used as the destination vector to synthesize TALEN mRNA. TALEN mRNA was transcribed from linearized plasmids using the mMessage mMachine T3 Transcription kit (Thermo Fisher Scientific, Cat#AM1348).

Morpholino and TALEN Injections

Initially, 4.5 ng of each *t* (*Xbra*) and *t2* (*Xbra3*) splice- and translation-blocking morpholino oligomer (MO_{splice} and MO_{transl}) were injected at 1-cell stage. MOs were designed by Gene Tools (see Key Resources Table). While the standard control MO was untagged, all *Brachyury* MOs were tagged 3’ with sulforhodamine. In an attempt to minimize MO side effects, this dose was incrementally reduced to 1.5, 0.75 and 0.25 ng per target MO. The standard control MO (cMO) from Gene Tools was used as a control and dosed accordingly. Wild type outbred *X. tropicalis* embryos were injected at the 1-cell stage with either 300 pg (*t*) or 600 to 1200 pg (*t2*) of TALEN mRNA. The heterozygous *X. tropicalis* lines $t^{e1.2D/+}$ and $t^{e1.2D/+}t2^{e3.7D/+}$ were submitted to the European *Xenopus* Research Centre (EXRC, https://xenopusresource.org).

Genotyping

Whole embryos or clipped tails from anaesthetized embryos (Love et al., 2013) were digested in 60 µl lysis buffer (50 mM Tris pH 8.5, 1 mM EDTA, 0.5% [v/v] Tween-20 and 100 µg/ml proteinase K [Thermo Fisher Scientific, Cat#AM2548]) for 2 hrs at 55°C. The digest was incubated for 10 mins at 95°C to inactivate proteinase K and spun briefly prior to PCR amplification. 2 µl of the lysate were used for each PCR reaction together with 200 nM of each forward and reverse primers (Key Resources Table) and KAPA HiFi HotStart ReadyMix (Kapa Biosystems, Cat#KK2602) in a 10 µl reaction. The targeted site of mutagenesis was amplified under the following PCR cycling conditions: 45 secs 98°C, 36 cycles (10 secs 98°C, 10 secs 58°C (*t*) or 63°C (*t2*), 10 secs 72°C) and 20 secs 72°C. PCR reactions were directly digested with either SacI (*t*) or EcoRI (*t2*) and separated by gel electrophoresis. For genotyping of single embryos, which were collected in TRIzol (Thermo Fisher Scientific, Cat#15596026) for total RNA extraction, genomic DNA was extracted according to the manufacturer’s back extraction protocol. For Sanger sequencing PCR products were purified and either sequenced directly or after TOPO TA cloning (Thermo Fisher Scientific, Cat#K4500J10).

Cloning of Wild-Type and Mutant *t* and *t2*

N- and C-terminal 3xHA tagged *t*, *t2*, $t^{e1.2D}$ and $t2^{e3.7D}$ constructs were created by Gateway cloning. The coding sequence (CDS) of *t*, $t^{e1.2D}$ and $t2^{e3.7D}$ was synthesized *de novo* by Thermo Fisher Scientific, while the CDS of *t2* was generated from the IMAGE cDNA clone 5307982 (Source BioScience). These sequences were PCR-amplified and inserted unidirectionally into the pENTR/TOPO entry vector (Thermo Fisher Scientific, Cat#K240020). The primers are listed in Key Resources Table. The entry vectors were recombined with N- or C-terminal 3xHA pCS2+ destination vectors. The final constructs were linearized with *Apa*I for *in vitro* transcription with the mMessage mMachine SP6 Transcription kit (Thermo Fisher Scientific, Cat#AM1340). Likewise, capped mRNA was generated from the injection control construct pCS2+ containing myc-tagged *fam83g* (kindly provided by Kevin Dingwell) linearized with *Pvu*II. 400 pg of each mRNA was injected into the zygote of *X. laevis* for its overexpression.

Western Blotting

X. laevis embryos were homogenized in 6 μl per embryo of PhosphoSafe extraction buffer (Merck, Cat#71296) supplemented with complete EDTA-free protease inhibitors (Sigma, Cat#11873580001). To remove yolk from the embryonic extract, the homogenate was mixed with the same volume of Freon (1,1,2-trichloro-trifluoroethane) and centrifuged for 5 mins at 10,000 g (4°C). One embryo equivalent of denatured supernatant was loaded onto pre-cast SDS-polyacrylamide gels (any kD mini-PROTEAN TGX) (Bio-Rad, Cat#4569033). Proteins were separated by molecular weight (SDS-PAGE) and processed for conventional western blotting. The following primary and secondary antibodies were applied at the indicated dilutions in PBS containing 0.1% (v/v) Tween-20 and 5% (w/v) milk powder: 1:10,000 mouse monoclonal anti-HA (Sigma, Cat#H9658), 1:10,000 mouse monoclonal anti-c-Myc (Sigma, Cat#M5546), 1:5,000 mouse monoclonal anti-α-tubulin (Sigma, Cat#T5168) and 1:2,000 goat anti-mouse IgG (H+L) horseradish peroxidase conjugate (Thermo Fisher Scientific, Cat#31430). Peroxidase activity was detected by chemiluminescence (BioFX Chemiluminescent Ultra-Sensitive HRP Membrane Substrate, SurModics) and captured with a ChemiDoc XRS+ imager (BioRad).

Total RNA Isolation

For deep sequencing, individual embryos from three separate $t^{e1.2D/+}t2^{e3.7D/+}$ *x* $t^{e1.2D/+}t2^{e3.7D/+}$ crosses or individual embryos from three separate *in vitro* fertilizations injected with either *t* and *t2* or control MOs were collected at developmental stages 26 (mid-tabud) and 34 (late tailbud). They were then homogenized each in 200 μl TRIzol by vortexing. For phase separation, 40 μl of chloroform was added to the homogenate, which was shaken vigorously for 15 secs before spinning for 5 mins at 16,000 g at 4°C. The aqueous phase containing total RNA was snap-frozen in liquid nitrogen and stored at -80°C while the interphase-organic layer was processed to extract genomic DNA for genotyping as described above. The aqueous phases from 5 to 10 sibling embryos of shared genotype or treatment were then combined. Total RNA was precipitated with one volume of absolute ethanol and cleaned using the RNA Clean and Concentrator 5 or 25 (Zymo Research) with in-column 3 U Turbo DNase (Thermo Fisher Scientific, Cat#AM2238) treatment according to the manufacturer's instructions. For RT-qPCR of uninjected and morphant embryos, 6 embryos from three to four separate fertilizations were collected for each developmental stage and processed as described above except that volumes were adjusted to the homogenization in 400 μl TRIzol and no genomic DNA was extracted.

RNA-Seq Library Preparation and Sequencing

Poly(A)+ RNA-Seq libraries were made from ~1 μg total RNA by following version 2 of the TruSeq RNA low sample protocol (Illumina) with the following modifications. After second strand synthesis 1 μl of the remaining eluate was used to measure the cDNA concentration using the Qubit fluorometer and Qubit dsDNA high sensitivity reagents (Thermo Fisher Scientific, Cat#Q32851). The resultant cDNA yield was used to estimate the number of requisite PCR cycles to generate high complexity libraries without chimera fragments. We routinely used 10 PCR cycles for 10 ng of cDNA and adjusted the number of PCR cycles accordingly. Libraries were sequenced on an Illumina HiSeq 4000 platform to produce paired-end reads of 76 bases. Read numbers and alignment statistics are summarized in Table S1.

RNA-Seq Differential Expression Analysis

Paired-end reads were aligned to a revised version of *X. tropicalis* gene models v7.2 and known off-genome EST assemblies including ribosomal and mitochondrial RNA (Collart et al., 2014) by running Bowtie2 v2.1.0 (Langmead and Salzberg, 2012) with the following constraints: -k 200 (up to 200 alignments per fragment) -X 800 (maximum fragment length of 800 bp) –rdg 6,5 (penalty for read gaps of length N, 6+N*5) –rfg 6,5 (penalty for reference gaps of length N, 6+N*5) –score-main L,-.6,-.4 (minimal alignment score as a linear function of the read length x, f(x) = -0.6 - 0.4*x) –no-discordant (no paired-end read alignments breaching maximum fragment length X) –no-mixed (only concordant alignment of paired-end reads). Only read pairs that uniquely align to one gene were counted. The reads of public RNA-Seq datasets (study accession # PRJNA351216, Campbell et al., 2016; PRJNA218018, Chung et al., 2014; PRJNA266550, Dichmann et al., 2015; PRJNA290093, Marlétaz et al., 2015; PRJEB8711, Noiret et al., 2016) were aligned to the genome assemblies of *X. tropicalis* (v7.1) or *X. laevis* (v9.1) using the STAR aligner v2.5.3a with default settings. Differential expression analysis was performed with raw fragment counts excluding those belonging to ribosomal and mitochondrial RNA using DESeq2 v1.14.1 (Love et al., 2014). Except for the principal component analysis (PCA) gene-specific dispersion estimates were calculated separately for the *t/t2* KO and KD experiment. In an effort to find genes with consistent fold changes over time, p-values were generated according to a likelihood ratio (χ^2) test reflecting the probability of rejecting the reduced (~ developmental stage) over the full (~ developmental stage + condition) model. Resulting p-values were adjusted to obtain false discovery rates (FDR) according to the Benjamini-Hochburg procedure, whereby thresholds on Cook's distances and independent filtering were switched off (Table S2). For PCA normalized fragment counts were transformed with a regularized logarithm (rlog) to shrink substantial variance among low-count genes (Figure 2A). For all other analyses, genes with ≤7 fragment counts averaged between cMO-injected and uninjected embryos and wild-type and *t/t2* heterozygotes were removed (Figure S2B). This lower threshold was set because markedly more falsely discovered fold changes were detected below it between otherwise very similar conditions. Public datasets were also analyzed using DESeq2, but statistical significance of transcript level differences between morphant and corresponding uninjected embryos were obtained through Wald tests (Figure 6A).

Perturbation Networks of Biological Processes

Common and unique changes ($\geq$1.5-fold change at FDR $\leq$10%) of gene expression among four comparisons (control or *t/t2* morphants versus uninjected embryos and *t/t2* heterozygotes or homozygotes versus wild-type embryos) were calculated and visualized in two Venn diagrams (Figure 2C) for elevated and reduced transcript levels using limma v3.30.13 (Ritchie et al., 2015). The five largest Venn fields were analyzed for enriched biological processes (BP) using GOstats v2.40.0 (Falcon and Gentleman, 2007) and GSEABase (Morgan et al., 2017). Gene-specific BP term associations were previously generated using BLAST2GO (Conesa et al., 2005; Gentsch et al., 2015; Owens et al., 2016). Parental, regulatory and component relationships between enriched BP were visualized as 'perturbation networks' using igraph v1.0.1 (Csárdi and Nepusz, 2006). Perturbation networks of Venn fields with similar BP signatures were joined (Table S4). The size of the node reflects the number of genes, while its color represents the hypergeometric p-value (-$\log_{10}$ p). Non-connected (0 degree) and low-grade (p >0.0001 or <10 genes for Figure 2E and Table S4 tabs 'DOWN_Venn_D' and 'UP_Venn_A,B+C' and <5 genes for all remaining tabs of Table S4) nodes were excluded from network drawing. Nodes were clustered into communities based on edge betweenness using the Newman-Girvan algorithm. For the purpose of visualization few of these communities were subsequently removed or merged manually. The network graph was drawn using the force-directed Fruchterman-Reingold algorithm. Some of the most enriched BP terms all of which have >100 universe members (GO:0006955, immune response; GO:0001816, cytokine production; GO:0007249, I-κB kinase/NF-κB signaling; GO:0001756, somitogenesis; GO:0072358, cardiovascular system development; GO:0003012, muscle system process; GO:0021510, spinal cord development; GO:0006520, cellular amino acid metabolism; GO:0006091, generation of precursor metabolites) were visualized by their hypergeometric p-value (-$\log_{10}$ p) in a bubble plot (Figure 2D).

Visualization of Chromatin and RNA Profiles

The local chromatin binding pattern of Brachyury (+/-40 kb from TSS) at tailbud stage was extracted from previously published ChIP-Seq data (Gentsch et al., 2013) and displayed alongside transcriptional fold changes for the most mis-regulated genes associated with the immune response or somitogenesis (Figure 3A). ChIP-Seq reads were aligned to the *X. tropicalis* genome assembly v7.1 using Bowtie2 v2.1.0 (Langmead and Salzberg, 2012) with default settings. The binding matrix for the heatmap (Figure 3A) was generated with a 500-bp resolution using HOMER v4.8.3 (Heinz et al., 2010). The number of uniquely aligned ChIP-Seq reads was normalized to the effective total of 10 million aligned reads including multimappers. Genomic regions which displayed <50% of the mean read density within a 400-bp window sliding through the input track by 200-bp increments were masked to eliminate any false positive enrichments. For visualizing *tp53* isoforms (Figure 3D) paired-end reads were mapped to the *X. tropicalis* genome assembly v7.1 and known off-genome EST assemblies using Tophat v2.0.10 (Kim et al., 2013) with the following parameters: -r 77 (mean inner distance between mate pairs) –mate-std-dev 110 (standard deviation of inner distances between mate pairs) -G v7.2 (gene models of version 7.2 as used above) -g 200 (up to 200 alignments per read) –report-secondary-alignments (include additional and secondary alignments). Tophat BAM files of biological replicates were merged using samtools v1.3.1 (Li et al., 2009) and converted to the bigWig format. These genome tracks were normalized to the wigsum of 1 billion excluding any reads with mapping quality <10 using the python script bam2wig.py from RSeQC v2.6.4 (Wang et al., 2012). Tracks were visualized in the IGV genome browser v2.3.92 (Robinson et al., 2011).

Analysis of Differential Splicing

Splicing anomalies were detected without transcript annotation applying LeafCutter v1.0 (Li et al., 2018) on STAR-aligned split reads from all conditions. LeafCutter focuses on intron splicing events rather than whole transcript isoform quantification which helped to reduce false positive accounts caused by whole transcript fold changes. The two-pass mode and otherwise default settings of STAR v2.5.2/3a were used to align split reads to the genome assemblies of *X. tropicalis* (v7.1) or *X. laevis* (v9.1) (Dobin et al., 2013). The scripts 'leafcutter_cluster.py' and 'utils.R' were modified accordingly to accept scaffold coordinates and to record all junctions that contain $\geq$1 read. LeafCutter clusters introns according to shared acceptor or donor sites. Here clusters with $\geq$7 reads and single introns having a maximum length of 0.5 Mb were selected: leafcutter_cluster.py –minclureads 7 –maxintronlen 500000. Differential splicing required all samples per condition to contain $\geq$7 supporting reads per junction. For visualization split read counts with more than one count per million (CPM) per splicing junction were TMM normalized using edgeR (Robinson and Oshlack, 2010; Robinson et al., 2010). Subsequently, only junctions with $\geq$20 split reads on average among all samples were kept. For each differentially spliced intron cluster (FDR $\leq$1%), only the splice events with the minimal ($\geq$1.5 standard deviations below the mean) and maximal percentage splice index (PSI) were represented in a heatmap (Figures 5C and 6B). MO sequence alignments to the genome and transcriptome were found by an exhaustive approach using the Bio.Seq module in Julia (https://github.com/BioJulia). The longest consecutive number of matching bases was calculated at each position of either strand of the genome, and at each position of the reverse strand of the transcriptome, recording positions with $\geq$8 consecutive matches and within 75 bp from putatively blocked splice sites. The heatmap values were reordered according to the differential use ($\log_2$ fold changes) of introns with a minimal PSI per cluster using Anti-Robinson seriation by simulated annealing (ARSA) (Hahsler et al., 2008). The Mann Whitney U test was applied to find out whether MO sequence alignments ($\geq$10 consecutive base matches) were enriched at mis-spliced junctions compared to all gene annotated splice junctions ($\geq$20 split reads detected among all samples) and whether negative transcript level changes were greater at mis-spliced genes than all other genes with confirmed introns ($\geq$20 split reads detected among all samples). Expected numbers of MO sequence alignments and negative fold changes ($\leq$67%) were determined by bootstrapping (n = 1,000). Read coverage across exons, introns and splice junctions were normalized and averaged across biological triplicates

(and developmental stages) to generate Sashimi plots using splAdder (Kahles et al., 2016). The consensus donor splice sequence (Figure 5B) was derived from annotated canonical splice junctions detected by $\geq$10 split reads across all control samples (uninjected and wild-type) from both tailbud stages. The sequence logo was generated from 145,447 donor splice junctions using WebLogo 3.5.0 (Crooks et al., 2004): weblogo -A rna -U probability -c classic.

Generation of Digoxigenin-Labeled RNA Probes

The DNA templates for generating *c3ar1* and *tp53inp1* whole mount *in situ* hybridization probes were PCR amplified from *X. tropicalis* embryonic stage 18 cDNA. The template for the *tp53* probe was amplified from *X. laevis tp53* pCS105 plasmid (Cordenonsi et al., 2007). All products of ~1 kb were amplified with the KAPA HiFi HotStart polymerase using the following PCR cycling conditions: 45 secs 98°C, 40 cycles (10 secs 98°C, 10 secs 63°C, 15 secs 72°C), 20 secs 72°C. The primer sequences are listed in Key Resources Table. Fresh PCR products or size-selected bands were zero-blunt cloned into the pCRII-TOPO vector (Thermo Fisher Scientific, Cat#450245). Identity and direction of insert was verified by restriction digest and Sanger sequencing. Plasmids were linearized by restriction digestion and purified using the QIAquick PCR purification kit (Qiagen, Cat#28104). All *in situ* hybridization probes were transcribed from ~1 μg of linearized plasmid using 1x digoxigenin-11-UTP (Roche, Cat#11277065910), 40 U RiboLock RNase inhibitor (Thermo Fisher Scientific, Cat#EO0381), 1x transcription buffer (Roche, Cat#11465384001) and SP6 or T7 RNA polymerase (Roche, Cat#11487671001 or 10881767001) in a 20 μl reaction for 2 hrs at 37°C. The probe was treated with 2 U Turbo DNase (Thermo Fisher Scientific, Cat#AM2238) to remove the DNA template and was either purified by spin-column chromatography (Clontech, Cat#636089) or LiCl precipitation. The RNA was quantified with a Nanodrop spectrometer, diluted to 10 ng/μl (10x stock) with hybridization buffer (50% [v/v] formamide, 5x SSC, 1x Denhardt's, 10 mM EDTA, 1 mg/ml torula RNA, 100 μg/ml heparin, 0.1% [v/v] Tween-20, 0.1% [w/v] CHAPS) and stored at -20°C. The following plasmids, restriction enzymes and RNA polymerases were used for plasmid linearization and *in vitro* transcription to generate sense (data not shown) and antisense probes: *actc1* antisense, *X. laevis actc1* pSP21 (Mohun et al., 1984), *EcoR*I, SP6; *cav1* antisense, *X. tropicalis cav1* (Gentsch et al., 2013), *Bgl*II, T7; *c3ar1* antisense, *X. tropicalis c3ar1* pCRII-TOPO (this study), *BamH*I, T7; *c3ar1* sense, *X. tropicalis c3ar1* pCRII-TOPO (this study, not shown), *Not*I, SP6, *hoxd8* antisense, *X. tropicalis hoxd8* pCR2.1-TOPO (Gentsch et al., 2013), *Hind*III, T7; *myh6* antisense, *X. tropicalis myh6* pCRII-TOPO (Abu-Daya et al., 2009), *Xho*I, SP6; *tal1* antisense, *X. laevis tal1* pGEM-7Zf+ (EXRC), *Xho*I, SP6; *tbx6* antisense, *X. laevis tbx6* pBluescript KS- (Uchiyama et al., 2001), *Not*I, T7; *tp53* antisense, *X. tropicalis tp53* pCRII-TOPO (this study), *BamH*I, T7; *tp53* sense, *X. tropicalis tp53* pCRII-TOPO (this study, not shown), *Not*I, SP6; *tp53inp1* antisense, *X. tropicalis tp53inp1* pCRII-TOPO (this study), *Hind*III, T7; and *tp53inp1* sense, *X. tropicalis tp53inp1* pCRII-TOPO (this study, not shown), *Not*I, SP6.

Whole Mount *In Situ* Hybridization

Whole mount *in situ* hybridization (WMISH) was conducted using digoxigenin-labeled RNA probes. It was based on previously published protocols (Monsoro-Burq, 2007; Sive et al., 2000). *X. tropicalis* embryos were fixed with 1 ml of MEMFA (1x MEM, 3.7% [v/v] formaldehyde) in 5 ml glass vials for 1 h at room temperature. The embryos were then washed once in 1x PBS and 2 to 3 times in ethanol. Fixed and dehydrated embryos were kept at -20°C for at least 24 hrs to ensure proper dehydration before starting with the hybridization. Dehydrated embryos were washed once more in ethanol before rehydrating them in two steps to PBT (1x PBS, 0.1% [v/v] Tween-20). Embryos were treated with 5 μg/ml proteinase K (Thermo Fisher Scientific, Cat#AM2548) in PBT for 6 to 8 mins, washed briefly in PBT, fixed again in MEMFA for 20 minutes and washed 3 times in PBT. Embryos were transferred into baskets, which are kept in an 8x8 microcentrifuge tube holder sitting inside a 10x10 slot plastic box filled with PBT. Baskets were built by replacing the round bottom of 2 ml microcentrifuge tubes with a Sefar Nitex mesh. This container system was used to readily process several batches of embryos at once. These baskets were maximally loaded with 40 to 50 *X. tropicalis* embryos. The microcentrifuge tube holder was used to transfer all baskets at once and to submerge embryos into subsequent buffers of the WMISH protocol. Next, the embryos were incubated in 500 μl of hybridization buffer (50% [v/v] formamide, 5x SSC, 1x Denhardt's, 10 mM EDTA, 1 mg/ml torula RNA, 100 μg/ml heparin, 0.1% [v/v] Tween-20, 0.1% [w/v] CHAPS) for 2 hrs in a hybridization oven set to 60°C. After this pre-hybridization step, the embryos were transferred into 500 μl of 1 ng/μl of digoxigenin-labeled probe preheated to 60°C and further incubated overnight at 60°C. The pre-hybridization buffer was kept at 60°C. The next day embryos were transferred back into the pre-hybridization buffer and incubated at 60°C for 10 mins. Subsequently, they were washed 3 times in 2x SSC/0.1% [v/v] Tween-20 for 10 mins at 60°C, twice in 0.2x SSC/0.1% [v/v] Tween-20 for 20 mins at 60°C and twice in 1x maleic acid buffer (MAB) for 5 mins at room temperature. Next, the embryos were treated with blocking solution (2% [w/v] Boehringer Mannheim blocking reagent in 1x MAB) for 30 mins at room temperature, and incubated in antibody solution (10% [v/v] lamb or goat serum, 2% [w/v] Boehringer Mannheim blocking reagent, 1x MAB, 1:2,000 Fab fragments from polyclonal anti-digoxigenin antibodies conjugated to alkaline phosphatase) for 4 hrs at room temperature. The embryos were then extensively washed 4 times in 1x MAB for 10 min before leaving them in 1x MAB overnight at 4°C. On the final day of the WMISH protocol, the embryos were washed another 3 times in 1x MAB for 20 mins and equilibrated to working conditions of alkaline phosphatase (AP) for a total of 10 mins by submerging embryos twice into freshly prepared AP buffer (50 mM MgCl2, 100 mM NaCl, 100 mM Tris pH9.5, 1% Tween-20). At this stage, the embryos were transferred to 5 ml glass vials for monitoring the progression of the AP-driven colorimetric reaction. Any residual AP buffer was discarded before adding 700 μl of freshly prepared staining solution (AP buffer, 340 μg/ml nitro-blue tetrazolium chloride, 175 μg/ml 5-bromo-4-chloro-3'-indolyphosphate). The colorimetric reaction was developed at room temperature in the dark. Once the staining was clear and intense enough, the color reaction was stopped by 2 washes in 1x MAB. To stabilize and preserve morphological features, the embryos were fixed with Bouin's fixative without picric acid (9% formaldehyde, 5% glacial acetic acid)

for 30 mins at room temperature. Next, the embryos were washed twice in freshly prepared 70% ethanol/PBT to remove the fixative and residual chromogens. After rehydration to PBT in two steps, the embryos were treated with bleaching solution (1% H_2O_2, 5% formamide, 0.5x SCC) overnight at 4°C in the dark. Finally, the embryos were washed twice in PBS before imaging them in PBS on a thick agarose dish by light microscopy.

Visualizing Apoptosis by TUNEL Staining

Terminal deoxynucleotidyl transferase (TdT) dUTP nick end labeling (TUNEL) was applied to detect the level and spatial distribution of apoptosis *in situ* (Figure S3A). This protocol was based on previous work (Hensey and Gautier, 1998; Trindade et al., 2003). The embryos were fixed and dehydrated as outlined above for WMISH. Unless otherwise stated, protocol steps were performed at room temperature and, particularly, washes were kept at least 5 mins long. Dehydrated embryos were rehydrated to PBT (PBS, 0.1% [v/v] Tween-20) in two steps. The tissue of embryos was permeabilized by washing 4 times in PBT. Next, embryos were rinsed twice in PBS and transferred into 2 ml round bottom microcentrifuge tubes. Positive control embryos were incubated in 100 μl 1x TURBO DNase buffer for 30 mins. 50 μl 1x TURBO DNase buffer were removed before adding 10 U of TURBO DNase and incubating for 20 mins. These embryos were rinsed twice in PBT and twice in PBS. Subsequently, positive control and all other embryos were incubated in 100 μl 1x Terminal deoxynucleotidyl Transferase buffer (100 mM K-cacodylate, pH7.2, 2 mM $CoCl_2$, 0.2 mM DTT) for 30 mins. 50 μl TdT buffer were removed before adding 7.5 U TdT (Thermo Fisher Scientific, Cat#EP0161) and 25 pmol digoxigenin-11-dUTP (Roche, Cat#11558706910). This labeling reaction was run overnight at room temperature. The following day, the embryos were incubated in 1 mM EDTA/PBS for 1 hr at 65°C to inactivate TdT. The embryos were washed 4 times in PBT and pre-incubated in PBT/lamb serum (5:1) for 30 mins. Next, the embryos were incubated in fresh PBT/lamb serum (5:1) and 1:2,000 anti-digoxigenin conjugated to AP (Roche, Cat#11093274910) for 4 hrs. Embryos were washed twice in PBT before keeping them overnight in PBT at 4°C. The following day, embryos were washed 4 times in PBT for 15 mins and twice in AP buffer for 5 mins before initiating the staining reaction with freshly prepared staining solution (see WMISH protocol). After 10 mins (positive control embryos) to 40 mins at room temperature the color reaction was terminated by 2 washes with PBT. Embryos were fixed and bleached as outlined in the WMISH protocol.

Quantification of Transcription and Splicing

Approximately 750 ng total RNA was reverse transcribed with 40 U RNase H minus and point-mutant M-MLV reverse transcriptase (Promega, Cat#M3681), 500 μM of each dNTP and 10 μM random hexamers in a 10 μl reaction following this temperature regime: 15 mins at 25°C, 15 mins at 37°C, 45 mins at 55°C and 15 mins at 85°C. The RT reaction was subsequently diluted to 60 to 100 μl with molecular grade water for qPCR. 2 μl of the diluted RT reactions were amplified in technical duplicates with SYBR Green I master mix (Roche, Cat#04707516001) on a Light Cycler 480 II (Roche) cycling 55-times between 94, 60 and 72°C with each temperature step running for 10 secs and switching at +4.8°C/sec and -2.5°C/sec. At the end qPCR reactions were heated from 65 to 97°C with a gradual increase of 0.11°C/sec (melting curve) to ensure only fluorescence was collected from one specific amplicon. Figure 1B was based on absolute quantification while all other RT-qPCR results were normalized to *odc1* and uninjected embryos using the $2^{-\Delta\Delta Ct}$ method (Livak and Schmittgen, 2001). The threshold cycle (C_t) was derived from the maximum acceleration of SYBR fluorescence (second derivative maximum method). The PCR primers were designed to hybridize at ~60°C (T_m) and to generate 75 to 125 bp amplicons using Primer3 (Key Resources Table).

Measurement of Morpholino:RNA Hybridization Affinity and Kinetics

The affinities and kinetics of the hybridization between the *t* splice-blocking MO and various biotinylated RNA oligonucleotides (Figure 7D and Table S6) were measured assuming a simple 1:1 interaction on an Octet RED biolayer interferometer (Pall FortéBio). The RNA molecules were immobilized on streptavidin-coated biosensors at a concentration of ~10 μg/ml. The hybridization of the MO with the immobilized RNA was measured at 23°C and 35°C using 200 to 1,500 second association steps followed by 500 to 5,000 second dissociation steps. The buffer consisted of 10 mM phosphate (pH 7.4), 150 mM NaCl, 0.5 mg/ml bovine serum albumin and 0.005% Tween-20. The equilibrium dissociation constant K_d was determined from the instrument response against several MO concentrations ranging from 120 pM (canonical t_{target} RNA sequence) to 3.2 μM (scrambled $dtymk_{off\text{-}target}$ RNA sequence) using the method of least squares and independently from the ratio of the dissociation and association rate constants (k_{off}/k_{on}). The association phases were analyzed using the single exponential function $Y=Y_0+A(1-e^{-kobs*t})$ where Y was the level of binding at time t, Y_0 the binding at the start of association, A the asymptote, and k_{obs} the observed rate constant. k_{on} was determined as the slope of a plot of k_{obs} against the MO concentration. Similarly, k_{off} was determined by analyzing the dissociation phases with the formula $Y=Y_0+Ae^{-koff*t}$. The kinetic K_d was calculated as the ratio of k_{off} and k_{on}.

QUANTIFICATION AND STATISTICAL ANALYSIS

Error bars indicate the standard deviation (SD) derived from three to four biological replicates (n) for RT-qPCR quantifications. Sample sizes are indicated in the image or the associated figure legends. In Figure 1, two-sample homoscedastic, two-tailed t-tests were applied to determine whether divergence (≥1.5-fold change) from wild-type transcript levels are statistically significant. In Figures 3, 4, 5, and S4, two-tailed one-sample t-tests were calculated to determine whether ≥1.5-fold changes are significantly different from a hypothetical value of 1. The statistical significance of dosage-dependent and/or temperature effects in Figure 7 was based on

one- or two-way ANOVA tests with Sidak's correction for multiple comparisons and 90% confidence interval. The statistical significance of differential RNA-Seq was corrected for multiple comparison according to the Benjamini-Hochberg procedure. Asterisks in figures indicate (adjusted) p-values as follows unless otherwise stated: *, p<0.1; **, p<0.01; and ***, p<0.001.

DATA AND SOFTWARE AVAILABILITY

Raw RNA-Seq data (FASTQ files) and annotation files reported in this paper are available in the GEO database (www.ncbi.nlm.nih.gov/geo) under the accession number GEO: GSE96655. All analyses were performed in R v3.3.1 / Bioconductor v3.5, Perl v5.18.2, Python v2.7.12 or Julia v0.5 as described in the detailed methods.

Developmental Cell, Volume *44*

Supplemental Information

Innate Immune Response and Off-Target Mis-splicing

Are Common Morpholino-Induced Side Effects

in *Xenopus*

George E. Gentsch, Thomas Spruce, Rita S. Monteiro, Nick D.L. Owens, Stephen R. Martin, and James C. Smith

Legends for Supplemental Figures

Figure S1. Generation of the Double Heterozygous Line for *Brachyury* paralogues *t* and *t2*, Related to Figure 1

(A) Scheme to generate the $t^{e1.2D/+}t2^{e3.7D/+}$ ($t^{-/+}t2^{-/+}$) *X. tropicalis* line. (B,F) TALEN design for *t* or *t2* mutagenesis and positions of MOs blocking donor splice site (MO_{splice}) or translation initiation site (MO_{transl}) of the corresponding transcript. (C,G) TALEN-induced mutation rate at the targeted *Sac*I or *EcoR*I site as estimated by the partial restriction digest of specific PCR amplicons. (D) Sanger sequencing summary of generated indels in exon 1 of *t*. (E) Morphological defects caused by TALEN-induced *t* mutations at late tailbud stage. Scale bar, 0.5 mm. (H) Western blot of injected wild-type and mutant *t* or *t2* constructs tagged either N- or C-terminally with *HA*. The detection of exogenous myc (as part of the injected *fam83g-myc* mRNA) and endogenous α-tubulin were used as controls for injection/translation efficiency and gel electrophoresis loading, respectively. (I) Mutant *Brachyury* constructs failed to disrupt gastrulation. Scale bar, 0.25 mm.

Figure S2. *Brachyury* KO and KD Embryos Are Morphologically Very Similar, Related to Figure 2

(A) Gross morphological comparison between different conditions of the KD and KO experiment at the indicated tailbud stages. Uninjected (uni) and control MO (cMO)-injected embryos were controls for the *t/t2* morphants (*t/t2* MO). Wild-type (wt) embryos were siblings of the mutant embryos (hetero- and homozygous for the mutant *t* and *t2* allele, *t/t2* het and *t/t2* KO). Scale bar, 0.5 mm. (B) Comparison of low fragment count gene transcripts to estimate minimal fragment count required for calling reliable fold changes. Genes that averaged <7 fragments between cMO-injected and uninjected and heterozygous and wild-type embryos over tailbud stage 26 and 34 were excluded due to a higher degree of spurious fold changes.

Figure S3. Increased Transcription of *tp53* Depending on GC Content of MO Does Not Cause More Apoptosis, Related to Figure 3 and 4

(A) TUNEL assay on morphants and sibling embryos from double heterozygous $t^{-/+}t2^{+/-}$ parents. DNase-treated wild-type embryos were used as positive controls. (B) Single WMISH for *tp53 and* multi-probe WMISH for various mesoderm cell lineage and

derivative markers (*cav1*, notochord; *hoxd8*, pronephros; *myh6*, heart; *tal1*, ventral blood island; *tbx6*, paraxial mesoderm) of late tailbud embryos injected with single MOs or tracer sulforhodamine-dextran. Scale bar, 0.5 mm.

Figure S4. Specific MOs of the *t/t2* MO Cocktail Cause Off-Target Splicing Defects, Related to Figure 5

(A,C) Superimposed Sashimi plot of *abi1* and *bloc1s4* transcripts whose splicing was perturbed by the injected *t/t2* MO mix. Canonical and alternative splicing are shown with solid and dashed lines, respectively. Blocked splice sites containing matches of ≥8 consecutive bases with a specific MO are shown as alignments. Canonical Watson-Crick and non-canonical wobble base pairing are marked as vertical bar and colon, respectively. (B,D) RT-qPCR (n = 4, mean ± SD) confirmed that specific MOs of the *t/t2* MO mix were responsible for mis-splicing. The fold change ($\log_2$ scale) of transcript levels and alternative splicing between exon 7 and 11 (*abi1*) and exon 4 and 6 (*bloc1s4*) are shown as filled and solid bars, respectively. Two-tailed t-test: *, p ≤0.1; **, p ≤0.01. See Figure 3D and Key Resources Table for the design of RT-qPCR primers.

Figure S5. Temperature and MO Dosage Effects on *Brachyury* Phenotype and Immune Response Related Gene Transcription, Related to Figure 7

(A) WMISH for *tp53* of late tailbud embryos injected with 4.5 or 18 ng of the *t/t2* MO mix and developed at 22 ºC or 28.5ºC. *, Remark: Increasing incubation temperature also slightly up-regulated *tp53* in the absence of any MO (confirmed by RT-qPCR, data not shown). (B) Multi-probe WMISH for various mesoderm cell lineage and derivative markers (*cav1*, notochord; *hoxd8*, pronephros; *myh6*, heart; *tal1*, ventral blood island; *tbx6*, paraxial mesoderm) and single WMISH for *tp53, tp53inp1* and *c3ar1* of mid-tailbud (stage 26) and late tailbud embryos (stage 34) injected with 6 or 18 ng of the *t/t2* MO mix. White arrowheads point to the expression domains of *tbx6* and *cav1* that were not maintained in embryos without functional Brachyury. (C) WMISH for *tp53inp1* on wild-type (or *t/t2* heterozygous) and *t/t2* null mutant embryos as well as embryos injected with 1 and 3 ng MO. The embryos were developed to late tailbud stage 34 at 25-26ºC. Scale bar, 0.5 mm.

Figure S1

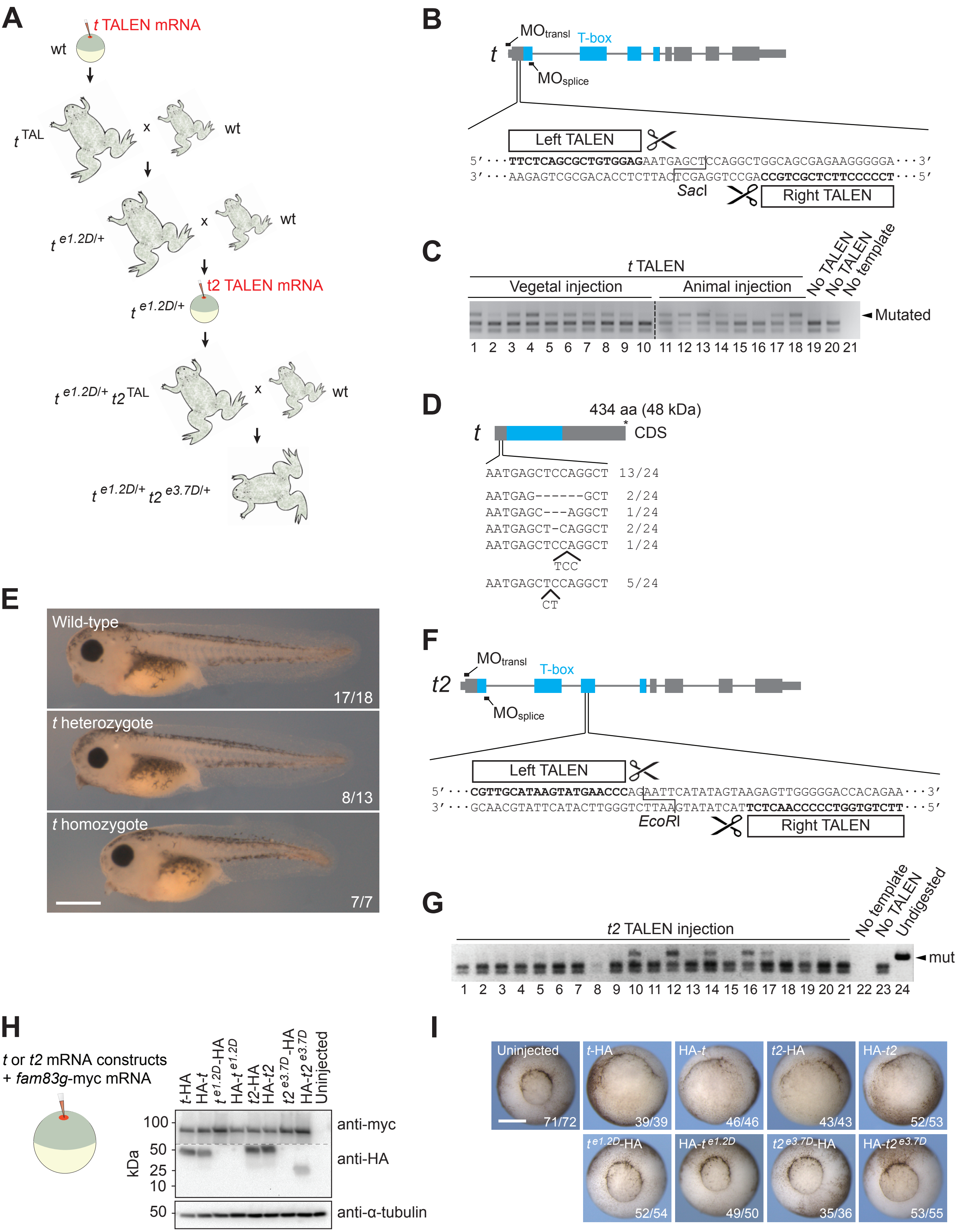
A
t TALEN mRNA
wt
t TAL
x
wt
t e1.2D/+
x
wt
t2 TALEN mRNA
t e1.2D/+
t e1.2D/+ t2 TAL
x
wt
t e1.2D/+ t2 e3.7D/+
B
MOtransl
T-box
t
MOsplice
Left TALEN
5′···TTCTCAGCGCTGTGGAGAATGAGCTCCAGGCTGGCAGCGAGAAGGGGGA···3′
3′···AAGAGTCGCGACACCTCTTACTCGAGGTCCGACCGTCGCTCTTCCCCCT···5′
SacI
Right TALEN
C
t TALEN
Vegetal injection
Animal injection
No TALEN
No TALEN
No template
Mutated
1 2 3 4 5 6 7 8 9 10 11 12 13 14 15 16 17 18 19 20 21
D
434 aa (48 kDa)
t
CDS
AATGAGCTCCAGGCT 13/24
AATGAG------GCT 2/24
AATGAGC---AGGCT 1/24
AATGAGCT-CAGGCT 2/24
AATGAGCTCCAGGCT 1/24
TCC
AATGAGCTCCAGGCT 5/24
CT
E
Wild-type
17/18
t heterozygote
8/13
t homozygote
7/7
F
MOtransl
T-box
t2
MOsplice
Left TALEN
5′···CGTTGCATAAGTATGAACCCAGAATTCATATAGTAAGAGTTGGGGGACCACAGAA···3′
3′···GCAACGTATTCATACTTGGGTCTTAAGTATATCATTCTCAACCCCCTGGTGTCTT···5′
EcoRI
Right TALEN
G
t2 TALEN injection
No template
No TALEN
Undigested
mut
1 2 3 4 5 6 7 8 9 10 11 12 13 14 15 16 17 18 19 20 21 22 23 24
H
t or t2 mRNA constructs
+ fam83g-myc mRNA
t-HA
HA-t
t e1.2D-HA
HA-t e1.2D
t2-HA
HA-t2
t2 e3.7D-HA
HA-t2 e3.7D
Uninjected
100
50
25
10
50
kDa
anti-myc
anti-HA
anti-α-tubulin
I
Uninjected
71/72
t-HA
39/39
HA-t
46/46
t2-HA
43/43
HA-t2
52/53
t e1.2D-HA
52/54
HA-t e1.2D
49/50
t2 e3.7D-HA
35/36
HA-t2 e3.7D
53/55

Figure S2

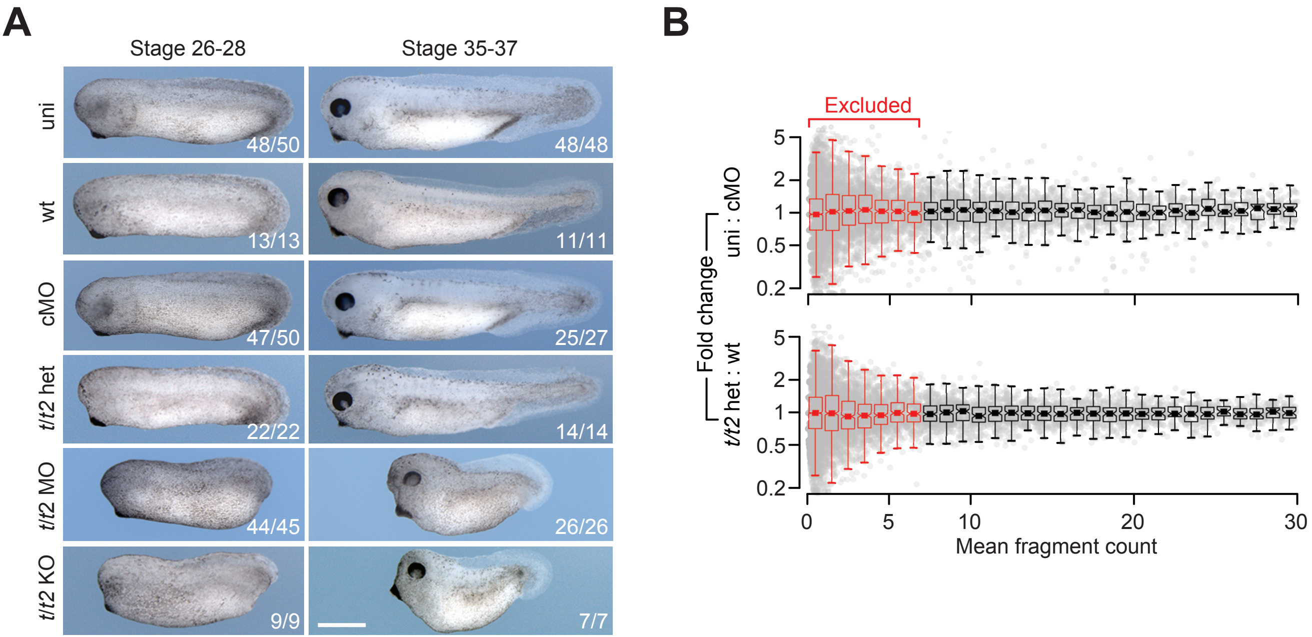
A
Stage 26-28
Stage 35-37
uni
wt
cMO
t/t2 het
t/t2 MO
t/t2 KO
48/50
48/48
13/13
11/11
47/50
25/27
22/22
14/14
44/45
26/26
9/9
7/7
B
Excluded
Fold change
uni : cMO
t/t2 het : wt
5
2
1
0.5
0.2
0
5
10
20
30
Mean fragment count

Figure S3

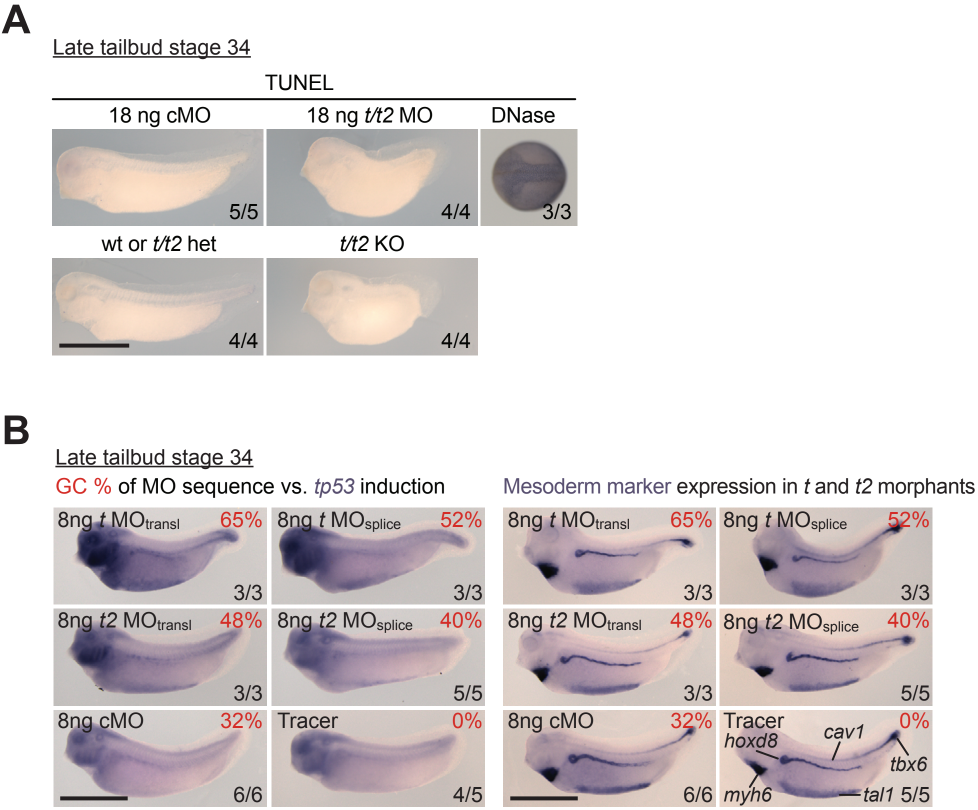
A
Late tailbud stage 34
TUNEL
18 ng cMO
18 ng t/t2 MO
DNase
5/5
4/4
3/3
wt or t/t2 het
t/t2 KO
4/4
4/4
B
Late tailbud stage 34
GC % of MO sequence vs. tp53 induction
Mesoderm marker expression in t and t2 morphants
8ng t MOtransl 65%
8ng t MOsplice 52%
3/3
3/3
8ng t2 MOtransl 48%
8ng t2 MOsplice 40%
3/3
5/5
8ng cMO 32%
Tracer 0%
6/6
4/5
8ng t MOtransl 65%
8ng t MOsplice 52%
3/3
3/3
8ng t2 MOtransl 48%
8ng t2 MOsplice 40%
3/3
5/5
8ng cMO 32%
6/6
Tracer 0%
hoxd8
cav1
tbx6
myh6
tal1 5/5

Figure S4

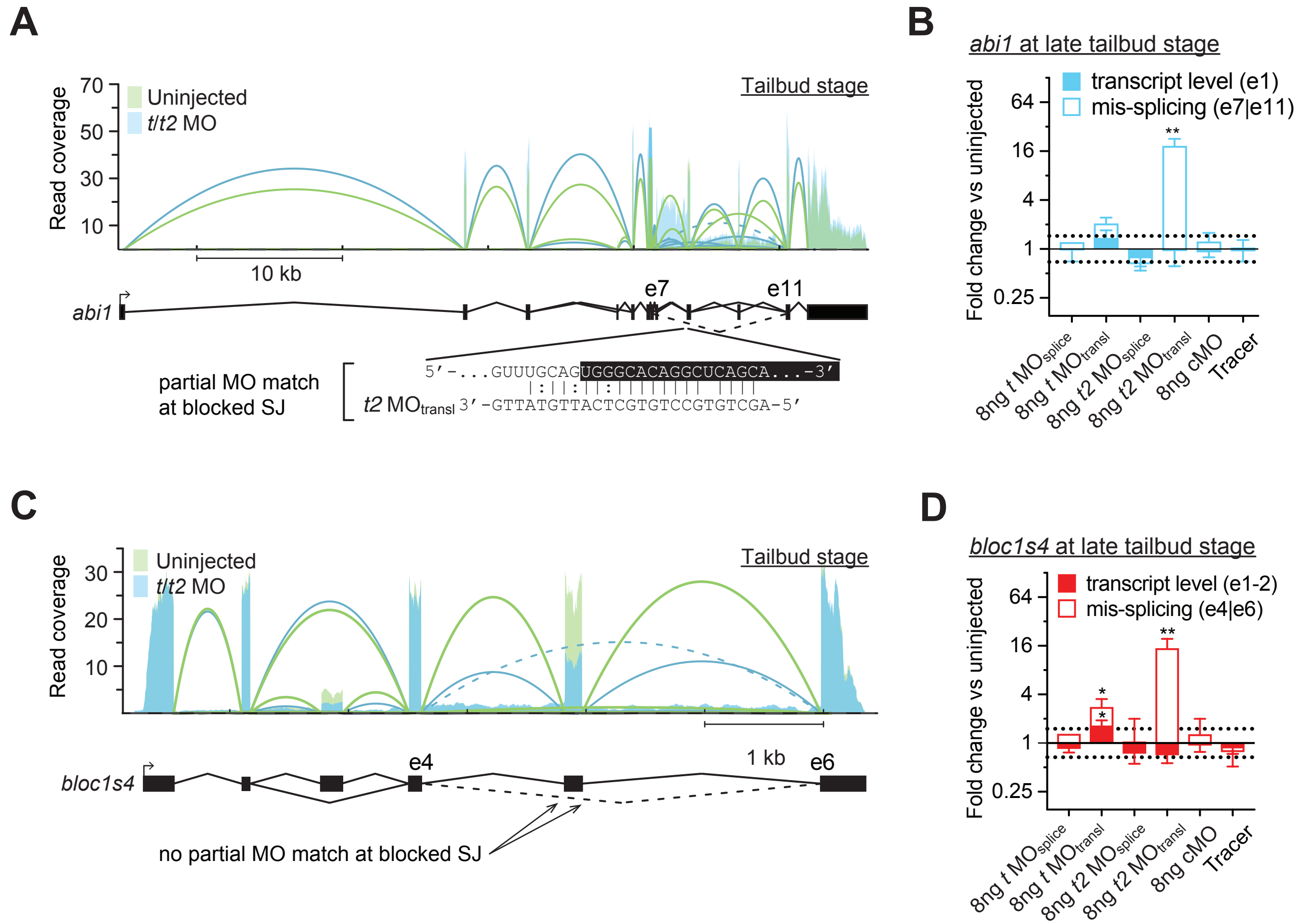
A
Read coverage
70
50
30
10
Uninjected
t/t2 MO
Tailbud stage
10 kb
e7
e11
abi1
partial MO match
at blocked SJ
5’-...GUUUGCAGUGGGCACAGGCUCAGCA...-3’
t2 MOtransl 3’-GTTATGTTACTCGTGTCCGTGTCGA-5’
B
abi1 at late tailbud stage
transcript level (e1)
mis-splicing (e7|e11)
Fold change vs uninjected
64
16
4
1
0.25
**
8ng t MOsplice
8ng t MOtransl
8ng t2 MOsplice
8ng t2 MOtransl
8ng cMO
Tracer
C
Read coverage
30
20
10
Uninjected
t/t2 MO
Tailbud stage
1 kb
e4
e6
bloc1s4
no partial MO match at blocked SJ
D
bloc1s4 at late tailbud stage
transcript level (e1-2)
mis-splicing (e4|e6)
Fold change vs uninjected
64
16
4
1
0.25
*
*
**
8ng t MOsplice
8ng t MOtransl
8ng t2 MOsplice
8ng t2 MOtransl
8ng cMO
Tracer

Figure S5

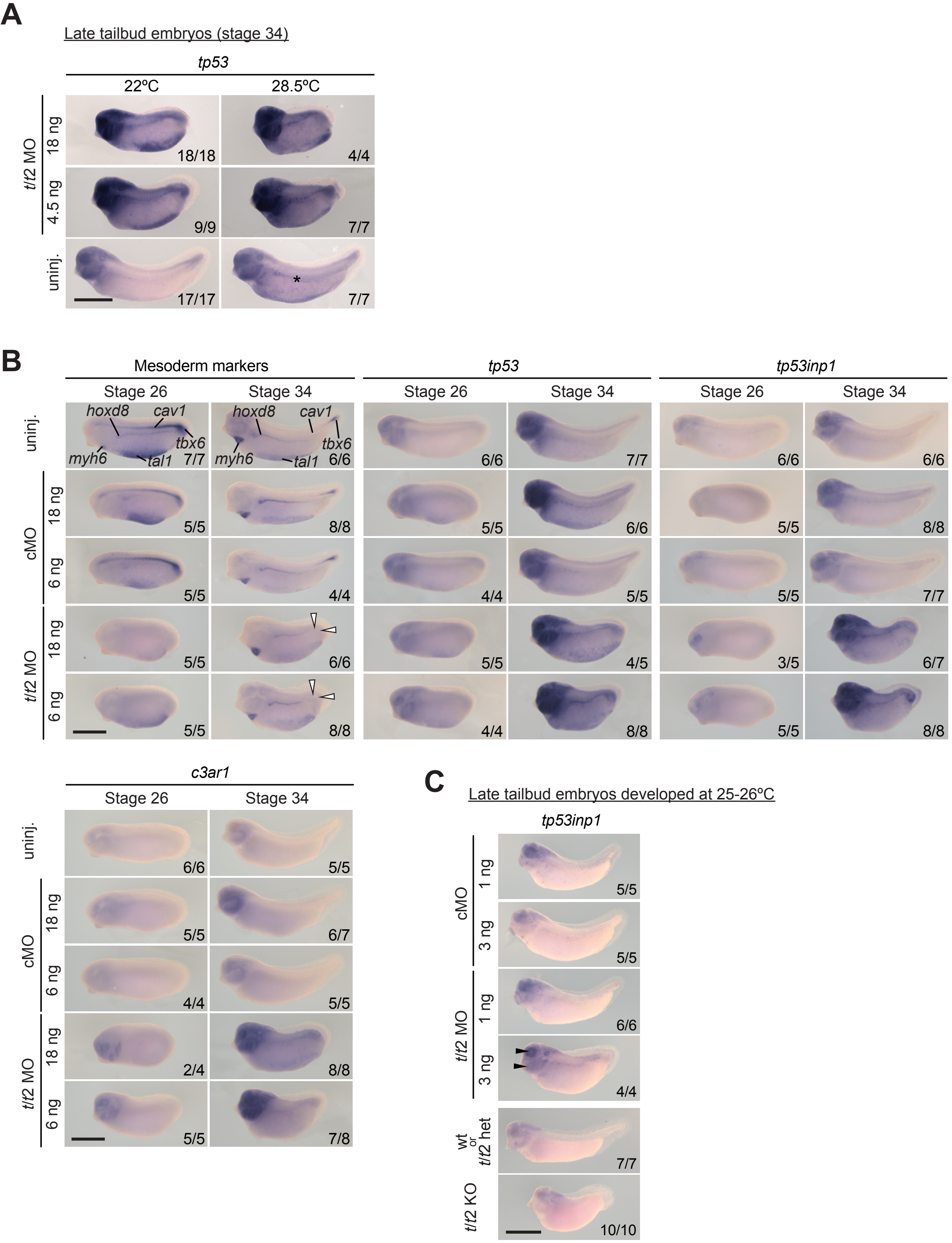

A
Late tailbud embryos (stage 34)
tp53
22ºC
28.5ºC
t/t2 MO
18 ng
4.5 ng
uninj.
18/18
4/4
9/9
7/7
17/17
7/7
*
B
Mesoderm markers
tp53
tp53inp1
Stage 26
Stage 34
hoxd8
cav1
tbx6
myh6
tal1
uninj.
cMO
18 ng
6 ng
t/t2 MO
7/7
6/6
6/6
7/7
6/6
6/6
5/5
8/8
5/5
6/6
5/5
8/8
5/5
4/4
4/4
5/5
5/5
7/7
5/5
6/6
5/5
4/5
3/5
6/7
5/5
8/8
4/4
8/8
5/5
8/8
c3ar1
Stage 26
Stage 34
uninj.
cMO
18 ng
6 ng
t/t2 MO
6/6
5/5
5/5
6/7
4/4
5/5
2/4
8/8
5/5
7/8
C
Late tailbud embryos developed at 25-26ºC
tp53inp1
cMO
1 ng
3 ng
t/t2 MO
5/5
5/5
6/6
4/4
wt or t/t2 het
7/7
t/t2 KO
10/10
